# Beneficial Effect of Edoxaban on Preventing Atrial Fibrillation and Coagulation by Reducing Inflammation *via* HBG1/HBD Biomarkers

**DOI:** 10.3389/fphar.2022.904317

**Published:** 2022-06-03

**Authors:** Chenguang Yang, Xiang Wang, Ying Guo, Xuyang Meng, Yi Li, Chenxi Xia, Lingbing Meng, Min Dong, Fang Wang

**Affiliations:** ^1^ Department of Cardiology, Beijing Hospital, National Center of Gerontology, Institute of Geriatric Medicine, Chinese Academy of Medical Sciences, Beijing, China; ^2^ Graduate School of Peking Union Medical College, Chinese Academy of Medical Science, Beijing, China

**Keywords:** atrial fibrillation, HBG1, HBD, edoxaban, inflammation, rivaroxaban

## Abstract

**Background:** Atrial fibrillation (AF) is the most common cardiac arrhythmia. The effectiveness and mechanism of edoxaban in preventing stroke after atrial fibrillation remain unclear.

**Methods:** The expressions of HBG1 and HBD in red blood cells were tested in AF. Sixty C57B/6J mice were randomly divided into the following groups: the control (CON) group, atrial fibrillation (AF) group, AF + edoxaban group, and AF + rivaroxaban group. H&E staining assay and reticular fiber staining were performed. Myocardial fibrosis was evaluated by the Masson staining assay, Sirius red staining assay, and immunohistochemical assay for the expressions of α-SMA and COL1A1. ELISA and RT-PCR assay were performed for the detection of inflammatory parameters (TNF-α, IL-1β, IL-6, and IL-10). Blood lipids were detected by using the Beckman automatic biochemical analyzer. Furthermore, four items of coagulation were detected, and molecular docking among HBG1, HBD, and MASP1 (Xa) was performed by PyMOL 2.1 software. The BP neural network model, cubic spline interpolation, and support vector machine model were constructed to predict prothrombin time based on HBG1 and HBD expressions. COIP assay was performed to construct the interaction between HBG1 and HBD. The functional enrichment analysis was performed by DAVID and Metascape tools.

**Results**: The expressions of HBG1 and HBD in red blood cells of the patients with atrial fibrillation were decreased. The results showed a lower level of hemoglobin in red blood cells with HBG1-siRNA and HBG1-siRNA. Compared with the AF group, the collagen fiber percentage in the AF + edoxaban group was decreased (*p* < 0.05). After using edoxaban, the expressions of TNF-α, IL-1β, IL-6, and IL-10 were significantly decreased (*p* < 0.05). The LDL-C, TC, and TG levels were downregulated in the AF + edoxaban group. The PT and APTT levels in the AF + edoxaban group were more increasing than in the AF mice (*p* < 0.05). Compared with the AF group, the expressions of HBG1 and HBD were downregulated in the AF + edoxaban group (*p* < 0.05). HBG1 protein matched well with HBD and MASP1(Xa) protein surfaces. There exists a significant interaction between HBG1, HBD, and PT *via* the BP neural network and support vector machine. Enrichment analysis showed that HBG1 and HBD were mainly enriched in blood coagulation.

**Conclusion:** Edoxaban could prevent atrial fibrillation and coagulation by reducing inflammation, lipids, and fibrosis *via* HBG1/HBD biomarkers effectively, and the effect was superior to that of rivaroxaban.

## Introduction

Atrial fibrillation (AF) is the most common cardiac arrhythmia (Miyasaka et al., 2006), affecting 33 million people worldwide annually (Chugh et al., 2013). AF is associated with a threefold-to-fivefold increased risk of stroke (Wolf et al., 1998), and AF-related strokes are typically severe, causing significant long-term physical disability, cognitive dysfunction, and high mortality and healthcare costs, compared to other stroke subtypes (Bruins et al., 1997; Kirchhof et al., 20162016). Since first reported by Bruins et al. (Bruins et al., 1997), there has been increasing evidence that the inflammatory status is closely related to the development of atrial fibrillation ((Katritsis 2006; Zhang et al., 2017; Zhou and Dudley 2020). Inflammatory responses, as evidenced by increased circulating levels of inflammatory mediators such as C-reactive protein (CRP), contribute to the persistence of AF (Bruins et al., 1997). Inflammatory mediators including ILs are thought to promote arrhythmogenesis as a result of structural and contractile remodeling of the atria and endocardium (Issac et al., 2007). The number of T lymphocytes and monocytes/macrophages in atrial myocardium was increased in AF patients (Smorodinova et al., 2017). With regard to thrombosis, elevated plasma CRP and interleukin (IL)-6 levels were independently associated with stroke risk (Lip et al., 2007; Roldán et al., 2012; Hu et al., 2015). Taken together, these studies suggest that patients with atrial fibrillation may benefit from the use of anticoagulants with anti-inflammatory properties or other aspects.

Edoxaban is a once-daily oral inhibitor of factor Xa, currently indicated for the prevention of stroke or systemic embolism in patients with nonvalvular AF (Giugliano et al., 2013). The protective effect might be explained by reduced factor Xa-mediated thrombin activation because thrombin triggers thrombus formation *via* activation of fibrinogen and platelets and induces inflammatory processes *via* protease-activated receptor (PAR) 1, 3, and 4 signaling (Popović et al., 2012).

HBG1 and HBD are important genetic components of hemoglobin β-globin (Patrinos et al., 2005). HBD has been found to be closely associated with inflammation (James et al., 2018), and upregulation of HBD has been observed during infection and inflammation (Özdemir et al., 2020). In agreement with previous studies, the GO annotations related to HBD are oxygen transport, iron ion binding, blood coagulation, and combination with oxygen (Chen et al., 2020). In previous studies, we found that HBG1/HBD may induce the occurrence and development of AF through inflammation and hemoglobin levels (Wang et al., 2022). We need further studies to evaluate the clinical implications of our results and whether patients with atrial fibrillation will benefit from the anti-inflammatory effects of edoxaban.

Therefore, in this study, AF model mice were used to explore the inhibitory effect of edoxaban on HBG1/HBD, which on the one hand reduced the degree of effect of AF on the myocardium and on the other hand plays a role in the inhibition of inflammation, lipids, and fibrosis. Meanwhile, we further compared the protective effects of edoxaban and rivaroxaban by inhibiting inflammation, lipids, and fibrosis.

## Methods

### Isolation of Red Blood Cells

The peripheral blood was taken from patients with and without AF using an anticoagulant tube. The sedimentation rate of normal peripheral blood erythrocytes and leukocytes is different, so it can be separated from normal peripheral blood erythrocytes. Anticoagulant venous blood was added to 3% gelatin brine or 6% dextran solution and then mixed. The tube is made to stand upright in a temperature box at room temperature or 37°C for 30–60 min. Gelatin and the red blood cell bonding effect make red blood cells sink quickly and white blood cells stay in the gelatin solution. A capillary pipette was used to absorb the upper layer of the fluid rich in white blood cells, which is then transferred to another tube. Ca^2+^, Mg^2+^ and Hank’s balanced salt solution were added to reach 3 cm from the mouth of the test tube and mixed well. The mixture was centrifuged at 2000 r/min for 10 min, and the supernatant was discarded and washed twice in the same way. The precipitated cells were suspended with Hank’s solution of 10–20% inactivated fetal calf serum and prepared with a suspension of desired cell concentration, usually 2 × 10^6^/ min.

### Immunofluorescence Assay of the Cell Smear

The cell suspension was centrifuged at 2800 rpm and 4°C for 5 min, the supernatant was discarded, and 2 ml of 4% paraformaldehyde was added to fix the cell suspension, according to the number of cells deposited at the bottom. The fixed cell suspension was centrifuged at 2,800 rpm/min and at 25°C for 5 min, the supernatant was discarded, and PBS was added according to the amount of sediment deposited at the bottom. The cell suspension was spread over the circle with a pipette placed to dry naturally. A measure of 50–100 μl of fixing solution was added inside the circle, and the serum was blocked. HBG1 protein was detected using a HBG1 polyclonal antibody (dilution rate = 1:1,000, 16824-1-AP; ProteinTech, Rosemont, United States). HBD proteins were detected using an HBD polyclonal antibody (dilution rate = 1:1,000, 25728-1-AP; ProteinTech, Rosemont, United States). The primary antibody was added overnight at 4°C. Cell climbing slides were covered with the secondary antibody for 50 min. The liquid was discarded slightly. Fluorescent microscopy was employed to observe the image. Positive cells are observed in red.

### Synthesis of HBG1-siRNA and HBD-siRNA and the Detection of Hemoglobin

The cells in the logarithmic growth phase were placed on 6-well plates with 5×10^5^ cells/well and transfected overnight. HBG1-siRNA and HBD-siRNA were constructed and obtained from the Beijing Qingke Biotechnology Co., Ltd. (Beijing, China). HBG1-siRNA and HBD-siRNA were transfected into peripheral blood erythrocytes by using the LipofectamineTM 3000 transfection reagent (Invitrogen, L300008). Before adding the transfection mixture, the liquid was drawn out from the 6-well plate and re-added to 2 ml fresh medium. A measure of 250 F06Dl of transfection mixture was added to the corresponding well, drop by drop, then shaken gently and mixed well. After 6-well plates were placed in a CO_2_ incubator for culturing, the cells were collected to extract RNA, and the expression levels of HBG1 and HBD were detected by qPCR. An automatic blood cell analyzer (Mindray, V500085) was used to detect the hemoglobin content.

### Construction of the Atrial Fibrillation Mouse Model

Sixty C57B/6J mice were randomly divided into the following groups: the control (CON) group, atrial fibrillation (AF) group, AF + edoxaban group, and AF + rivaroxaban group, with 15 mice in each group. The mice in the atrial fibrillation model group were intraperitoneally injected with 2.5 mg/kg/d isoproterenol (Southwest Taiji, 2 ml: 1mg, Southwest Pharmaceutical Co., Ltd.), and the mice in the blank control group were intraperitoneally injected with the same amount of normal saline. In order to increase the success rate of construction of the AF model, the high-fat diet was used. The ECG of mice was collected by using an electrocardiogram machine (VECG-230B, three sharp beasts) after 4 weeks of continuous injection. The control group had a standard Ⅱ lead electrocardiogram, and the mice in the modeling group had standard Ⅱ lead atrial fibrillation, suggesting that the atrial fibrillation model was successfully established. The AF + edoxaban and the AF + rivaroxaban group were given edoxaban 0.03 mg/d and rivaroxaban 0.01 mg/d on the next day after the successful modeling, respectively. The drugs were dissolved in the daily drinking water of mice and fed for 14 days. The blank control group and the atrial fibrillation model group were given the same amount of drinking water. After 14 days, the mice were killed, and their blood and heart tissue were collected.

### H&E Staining Assay

The heart tissue was paraffin-embedded and sectioned. The paraffin sections were dewaxed using water. The sections were stained with Harris’s hematoxylin for 3–8 min, washed with tap water, and differentiated with 1% of ethyl hydrochloric acid within seconds. Again, the sections were washed with tap water, which then returned to blue with 0.6% ammonia, followed by washing with tap water. The sections were stained with eosin solution (1–3 min). The slices were dehydrated, dried, and then sealed with neutral gum. Microscopic examination, image collection, and analysis were performed.

### Masson’s Staining Assay

The heart tissue was paraffin-embedded and sectioned. The paraffin sections were dewaxed with water. The slices were soaked in Masson’s A solution overnight and washed under running water. The solution was sectioned into Masson B solution and Masson C solution in an equal ratio mixture of dye for 1 min, washed with tap water, and differentiated with 1% hydrochloric acid alcohol, followed by washing with tap water. The slices were soaked in Masson’s D solution for 6 min and rinsed with tap water, followed by soaking in Masson’s E solution for 1 min. The slices were stained directly with Masson F solution for 2–30 s. Sections were rinsed and differentiated with 1% glacial acetic acid and dehydrated with two cylinders of anhydrous ethanol. Using the transparent sealing sheet, the slices were put into the third cylinder of anhydrous ethanol for 5 min and xylene for 5 min for sealing with transparent neutral gum. Microscopic examination, image collection, and analysis were performed.

### Sirius Red Staining Assay

The slice was dewaxed. The section was stained with Sirius red solution (8 min) and then dehydrated quickly with two or three cups of anhydrous ethanol. The microscope was used to observe the image and analysis. Under an optical microscope, it was observed that collagen fibers are red with a yellow background.

### Reticular Fiber Staining

Reticular fiber incubation solution is prepared with 2 ml of 10% silver nitrate; the concentrated ammonia was added drop by drop by shaking the container while adding, and then, the concentrated aqueous ammonia drop was added after precipitation occurs, until the precipitation is just dissolved. Then, 2 ml of 3% NaOH was added until the precipitate forms again, and the concentrated aqueous ammonia was added drop by drop, until the precipitation is just dissolved; ultra-pure water was added to the mixture to make up to 40 ml (Miyasaka et al., 2006). For paraffin section deparaffinization and rehydration, the slides were washed with xylene I for 20 min, xylene II for 20 min, absolute ethanol I for 5 min, absolute ethanol II for 5 min, and 75% alcohol for 5 min, followed by tap water washing (Chugh et al., 2013). For tissue acidification, a circle was drawn to enclose the tissue, and then acidification solution (0.5% potassium permanganate and 0.5% sulfuric acid mixed in 1:1 ratio) was added drop by drop to oxidize the tissue for 5 min. The slides were then washed for 10 s by transferring it into two cylinders of ultra-pure water, and the slides were dried to remove excess water (Wolf et al., 1998). For tissue bleaching, a drop of 2% oxalic acid was added to the tissues for 2 min, the slides were washed in ultra-pure water for 3 times, 5 s each, and the slides were dried to remove excess water (Kirchhof et al., 20162016). For mordant staining, a drop of the Gordon and Sweet’s staining solution F was added to the tissue for 15 min (light-proof), the slides were washed in ultra-pure water for 3 times, 5 s each, and the slides were dried to remove excess water (Bruins et al., 1997). For Gordon and Sweet’s incubation solution staining, a drop of the Gordon and Sweet’s incubation solution was added to the tissues for 5 min (light-proof), the slides were washed in ultra-pure water for 3 times, 5 s each, and the slides were dried to remove excess water (Zhang et al., 2017). For reduction, a drop of 10% neutral formaldehyde solution was added to the tissue for 3 min, the slides were washed in ultra-pure water for 3 times, 5s each, and the slides were dried to remove excess water (Katritsis 2006). For dehydration and sealing with neutral balsam, the slides were washed in absolute ethanol I for 5 min, absolute ethanol II for 5 min, absolute ethanol III for 5 min, dimethyl I for 5 min, and xylene II for 5 min. (Zhou and Dudley 2020). Then, microscopic examination, image collection, and analysis were performed. The reticular fibers are black in color, and the background is brownish yellow.

### Immunohistochemical Assay

The paraffin section was deparaffinized and rehydrated. Antigen retrieval was performed by citric acid (pH 6.0) antigen retrieval buffer. For the assay, 3% hydrogen peroxide was used to block the endogenous peroxidase activity (25 min). Then, 3% BSA was added for serum sealing (30 min). α-SMA protein was detected using a α-SMA polyclonal antibody (dilution rate = 1:2,000, 14395-1-AP; ProteinTech, Rosemont, United States). COL1A1 proteins were detected using a COL1A1 polyclonal antibody (dilution rate = 1:3,000, 67288-1-Ig; ProteinTech, Rosemont, United States). The primary antibody was added overnight at 4°C. Tissues are covered with the secondary antibody (50 min). The DAB chromogenic reaction was performed. The positive target is brownish yellow. The sections are counterstained with hematoxylin stain solution for about 3 min. A microscope (Nikon, E100) was used to observe the image.

### Detection of the mRNA Expression *via* RT-qPCR

RNA was extracted using the TRIzol reagent, as per the manufacturer’s instructions. RNA concentration and purity were detected by using a NanoDrop 2000 spectrophotometer. After the instrument is reset blank, 2.5 μL of RNA solution was taken to be measured on the testing base, and the sample arm was lowered to take the readings. The absorbance was tested using a piece of computer software. Reverse transcription was performed by using the Sensiscript RT Kit (G3330, Servicebio), and the reagent was mixed gently and centrifuged. The primers are shown in Table 1. The reverse transcription program conditions are as follows: 25°C for 5 min; 42°C for 30 min; and 85°C for 5 s. A 0.1-ml PCR plate was taken to prepare the reaction system as follows: 2 × SYBR Green qPCR Master Mix (None ROX), 7.5 µL; F/R primers, 1.5 µL; cDNA, 2.0 µL; and nuclease-free water, 4.0 µL. Then, PCR amplification was performed. In the first step, predegeneration was performed for 30 s under the temperature of 95°C. The second step included 40 cycles, and each cycle consists of degeneration for 15 s under the temperature of 95°C and annealing/extension for 30 s under the temperature of 60°C. In the third step, the melting curve method was performed. Also, in this step, the temperature was changed from 65 to 95°C, and the fluorescence signal was collected at a 0.5 temperature rise. The relative quantitative expression data of genes were analyzed by the 2^−ΔΔCt^ method.

**TABLE 1:** Primers of the genes.

Gene name	Primer (5′-3′)
HBG1-S	ATG​GGT​CAT​TTC​ACA​GAG​GAG​G
HBG1-A	ATG​GGT​AGA​CAA​CCA​GGA​GCC
HBD-S	TGC​CTT​TAG​TGA​TGG​CCT​GG
HBD-A	AAC​AGT​CCA​GGA​TCT​CAA​TGG​T
α-SMA-S	GTC​TCA​GTC​AGC​CTA​AGG​AAG​CC
α-SMA-A	GAG​AAA​TGT​TGG​GCA​AAG​GGA
COL1A1-S	CAG​CAG​TAG​CCC​AGA​AGA​CAG​T
COL1A1-A	GGC​ATT​TCA​TAA​GCC​TCA​TTG​TC
TNF-α-S	TGGAGGGCTAGGATTTGG
TNF-α-A	TGG​TAG​GAG​ACG​GCG​ATG​C
IL-1β-S	AAACAAAGAAGGCTGGAA
IL-1β-A	GGT​GGC​TAA​GAA​CAC​TGG​A
IL-6-S	CCAACTTGTCGCACTCAC
IL-6-A	CTGCACTCTTGCCCTTGT
IL-10-S	GCTCCGCAGAAAGAAGAC
IL-10-A	TCAAAGCGAAGGAAACAA

### ELISA for the Detection of Inflammatory Parameters

The inflammatory biomarkers TNF-α, IL-1β, IL-6, and IL-10 were detected using the Mouse TNF-α Simple Step ELISA^®^ Kit (ab208348, Abcam, United States), Mouse IL-1β Simple Step ELISA^®^ Kit (ab197742, Abcam, United States), Mouse IL-6 Simple Step ELISA^®^ Kit (ab222503, Abcam, United States), and Mouse IL-10 Simple Step ELISA^®^ Kit (ab100697, Abcam, United States), respectively, as per the manufacturer’s instructions. The following steps were carried out to detect the inflammatory biomarkers using ELISA.

The buffer was encapsulated with carbonate; the antibody was diluted to a protein content of 1–10 μg/ml. A measure of 100 μL was added to each well of the polystyrene plate overnight at 4°C. Then, 200 F06Dl sealing solution was added to each well and incubated at 37°C for 1–2 h. The sealing plate film was removed carefully, placed into the washing machine, and washed for 3–5 times. Thereafter, 100 μL of the appropriately diluted sample was added to the coated reaction well. After sealing with a sealing plate membrane, it was incubated at 37°C for 1–2 h. Then, 100 μL of diluted biotinylated antibody working solution was added to each well. After sealing with a sealing plate membrane, it was incubated at 37°C for 1 h. It was followed by addition of 100 μL of the diluted enzyme conjugate to each well. After sealing the plate with sealing plate membrane, the plate was incubated at 37°C away from light for 30 min. Then, 100 μL TMB substrate solution was added to each well. At 37°C, the reaction was protected from light for 10–30 min until a distinct color gradient in the multiple dilution standard hole was found. Furthermore, 100 μL of 2M sulfuric acid was added to each reaction well, and the color changes from blue to yellow within 10 min on the microplate reader at 450 nm. The OD value of each hole was measured after adjusting the blank control hole. According to the standard concentration and OD value of the standard curve, the sample concentration was calculated.

### Determination of Blood Lipid

Whole blood samples were placed at room temperature for 2 h and separated at 2–8°C at 3000 rpm for 15 min. The supernatant was taken for packaging, and the specimen was stored at −80°C. The thawed samples were centrifuged again and then tested for blood lipids. Total cholesterol (TG), triglyceride (TG), low-density lipoprotein cholesterol (LDL-C), and high-density lipoprotein cholesterol (HDL-C) were determined by the Beckman automatic biochemical analyzer.

### Detection of Four Items of Coagulation

The mouse blood was slowly injected into a plastic tube containing 0.5 ml of 109 mmol/L sodium citrate solution and thoroughly mixed. The platelets were removed, and the plasma was separated by centrifugation at 3,000 rpm for 15 min. An automatic coagulation analyzer is used (Redu Life Science Co., Ltd., RAC-030) for the determination of four coagulation items: prothrombin time (PT), activated partial prothrombin time (APTT), thrombin time (TT), and fibrinogen content (FIB). The kits used in this experiment are as follows: prothrombin time (PT) kit (Batch No: 105090), Laibo Institute of Biological Experimental Materials; activated partial thrombin time (APTT) kit (Batch No. 111021), Laibo Institute of Biological Experimental Materials; thrombin time (TT) kit (Batch No.: 111027), Laibo Institute of Biological Experimental Materials; and fibrinogen (FIB) kit (Batch No.: 1110131), Beijing Laibo Institute of Biological Experimental Materials.

### Molecular Docking Among HBG1, HBD, and MASP1 (Xa)

HBG1 (PDB ID:1I3D), HBD (PDB ID : 1SHR), and MASP1 (PDB ID : 4KKD)target protein structures were obtained from the RCSB database (https:// www.rcsb.org/). All protein structures were processed in the molecular operating environment (MOE 2019.1). The position selected was Amber10, including the removal of water and ions, protonation, addition of missing atoms and completion of missing groups, and protein–energy minimization. Using HDOCK, the protein is set to rigid. The docking contact site is set to the full surface, and the conformation generated after docking is set to 100. The most negative conformation was selected by the scoring function and visualized by the PyMOL 2.1 software.

### Construction of the BP Neural Network Model and Cubic Spline Interpolation to Predict Prothrombin Time Based on HBG1 and HBD Expressions

Through the training of sample data, the BP neural network constantly revises the network weights and thresholds to make the error function descend along the negative gradient direction and approach the desired output. The model takes HBG1 and HBD expressions of each group of data as input and prothrombin time as output, so the number of nodes in the input layer is 2 and the number of nodes in the output layer is 1. A neural network with a hidden layer can approach a nonlinear function with arbitrary precision as long as there are enough hidden nodes. Therefore, a three-layer multi-input single-output BP network with a hidden layer is used to build a prediction model. In addition, the S-type tangent function tansig was selected as the excitation function of hidden layer neurons in this study. Since the output of the network is within the range of [−1, 1], the prediction model selects the S-type logarithmic function tansig as the excitation function of the neurons at the output layer. The neural network toolbox in MATLAB (MathWorks, 2017a) was used for network training. HBG1, HBD, and prothrombin time were quantified by cubic spline interpolation.

### Construction of the Support Vector Machine Model Based on HBG1, HBD, and PT

The support vector machine (SVM) can improve the generalization ability of the learning machine as much as possible. Even if the discriminant function is obtained from the limited data set, it can still get a small error for an independent test set. In addition, the support vector machine is a quadratic optimization problem, which can ensure that the extremum solution is the global optimal solution. SVM uses the hinge loss function to calculate empirical risk and adds a regularization term to the solving system to optimize structural risk. It is a classifier with sparsity and robustness. SVM, one of the common kernel learning methods, can be used for nonlinear classification by the kernel method. This study intends to use the support vector machine algorithm to build the correlation model between HBG1, HBD and PT.

### COIP Assay

Peripheral blood erythrocytes were isolated from mice for cell culture. An appropriate amount of the pre-cooled IP cell lysate was added to the cell culture dish, and the cells were lysed at 4°C for 10 min. During this period, the cells were repeatedly blown with a pipette, and then, the cell suspension was transferred to a 1.5-ml centrifuge tube for ice lysing for 20 min. After centrifugation at 12,000 r/min at 4°C for 10 min, the supernatant was transferred into a new 1.5-ml centrifuge tube, and the protein concentration was determined by the BCA method. A small amount of supernatant was taken for input experiment after denaturation, that is, WB detection of target protein. Then, 1.0 μg IgG and 20 μL protein A/G beads were added to the negative control (IgG) group protein supernatant. The experimental group was directly added with 20 μL protein A/G beads, and the mixture was shaken at 4°C and incubated for 1 h. Centrifugation was performed at 4°C for 5 min, and the supernatant was taken. 1–10 μL (0.2–2 μg) of antibody was added to the mixture and incubated overnight at 4°C. Thereafter, 80 μL protein A/G-beads were added, which were gently folded with fingers and incubated at 4°C for 2 h. Centrifugation was performed at 4°C for 5 min, the supernatant was carefully discarded, and the immunoprecipitation complex was collected. After the last washing, the supernatant was removed as much as possible; then, 80 μL of 1 × reduced sample loading buffer was added and boiled for 10 min. The supernatant was centrifuged at 4°C for 5 min, and the 10 μL supernatant sample was taken for Western blotting detection.

### Exploration of the Protein–Protein Interaction Network and Core Modules of HBG1- and HBD-Related Genes

HBG1- and HBD-related gene columns are searched in the Comparative Toxicogenomics Database (CTD). STRING software (version 10.5) was used to study the network of interactions between proteins, helping to mine core regulatory genes. There are many databases of protein interactions, and STRING is the largest of them. A single input of HBG1 and multiple proteins related to HBD will provide the interaction network between the input proteins, which is more suitable for mining the interaction between the input proteins. Each node represents a protein. The lines between the nodes represent the interaction between two proteins.

Specific parameters in the protein–protein interaction (PPI) network analysis were followed.

Network type: full STRING network (the edges indicate both functional and physical protein associations).

Meaning of network edges: evidence (line color indicates the type of interaction evidence).

Active interaction sources: textmining; experiments; databases; co-expression; neighborhood; gene fusion; co-occurrence.

Minimum required interaction score:0.150.

Max number of interactors to show: none.

Network display mode: interactive svg (network is a scalable vector graphic [SVG]; interactive).

Cytoscape software (V3.8.0) was used to visualize the network. Cytoscape is an open source web software that helps users build networks by integrating, analyzing, and visualizing data. The software comes with an editor module that allows users to set up networks directly within the software. MCODE plug-in and cytoHubba were used to find active core modules in the PPI network. According to the gene expression data, the network was screened to find the interaction connectome, that is, the interaction subnetwork, whose genes showed particularly high differential expression level.

### Functional Enrichment Analysis of HBG1- and HBD-Related Genes

GO (gene ontology, http://geneontology.org/, GO release date: 2019–01–01 and “doi:10.5281/zenodo.2529950”) annotates gene products in terms of function, participating biological pathway, and localization in cells. KEGG (Kyoto Encyclopedia of Genes and Genomes, https://www.genome.jp/kegg/pathway.html, version: 24 March 2022) is a comprehensive database integrating genomic, chemical, and systematic functional information. The Database for Annotation, Visualization, and Integrated Discovery (DAVID, version 6.8) is a biological information database that integrates biological data and analytical tools, and provides systematic and comprehensive biofunctional annotation information for large-scale gene or protein lists. At present, the DAVID database is mainly used for functional and pathway enrichment analysis of differential genes. Metascape (version 3.5) integrates more than forty bioinformatics databases. It includes enrichment analysis of biological pathways, structural analysis of protein interaction networks, and abundant gene annotation capabilities, and presents the results in a high-quality graphic language that biologists can easily understand. In this study, DAVID and Metascape software were used to analyze the functional enrichment of HBG1- and HBD-related genes.

### Screening of the Small Molecule Compounds of HBG1- and HBD-Related Genes Based on the Comparative Toxicogenomics Database

Small molecule compounds of HBG1- and HBD-related genes were screened using the Comparative Toxicogenomics Database (CTD).

### Statistical Analysis

SPSS software, version 23.0 (IBM Corp., Armonk, NY, United States), was used for data statistics. Data are expressed as mean ± standard deviation. One-way ANOVA was used for the comparison between the four groups, and LSD and Dunnett’s test were used for pairwise comparison. Student’s *t* test was used for the comparison between the two groups. *p* < 0.05 (two-tailed) was considered statistically significant.

## Results

### Expressions of HBG1 and HBD in the Red Blood Cells of the Patients With Atrial Fibrillation

**FIGURE 1:**
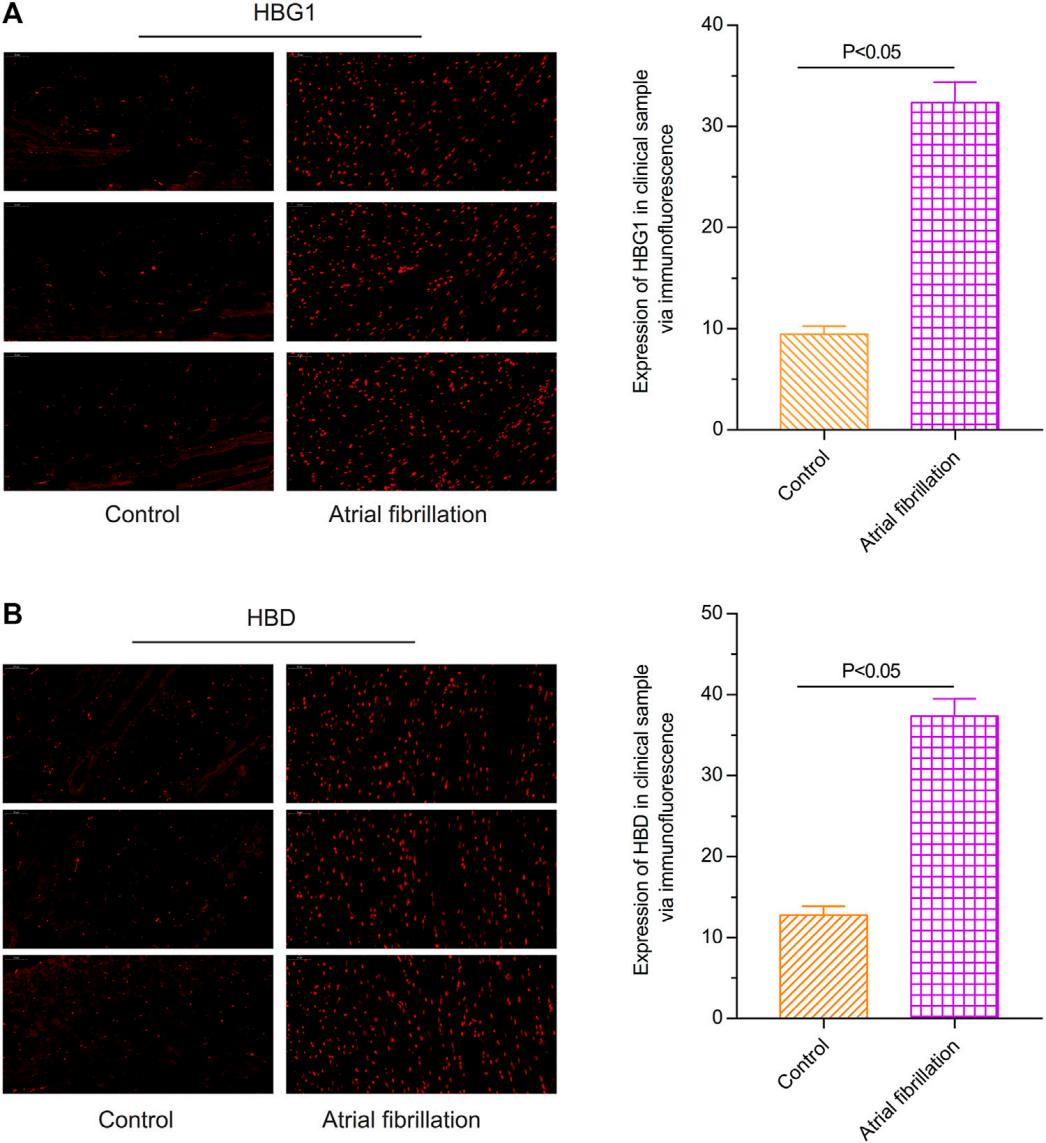
Expressions of HBG1 and HBD in the red blood cells of the patients with atrial fibrillation *via* the immunofluorescence assay. Compared with the red blood cells of the control individuals, the expressions of **(A)** HBG1 and **(B)** HBD were higher in the red blood cells of the patients with atrial fibrillation.

Through the immunofluorescence assay, compared with the red blood cells of the control individuals, the expression of HBG1 was higher in the red blood cells of the patients with atrial fibrillation (Wang et al., 2022) (*p* < 0.05, Figure 1A). In addition, the expression of HBD was also higher in the red blood cells of the patients with atrial fibrillation than in those of the control individuals (*p* < 0.05; Figure 1B).

### Lower Level of Hemoglobin in the Red Blood Cells With HBG1-siRNA and HBG1-siRNA

**FIGURE 2:**
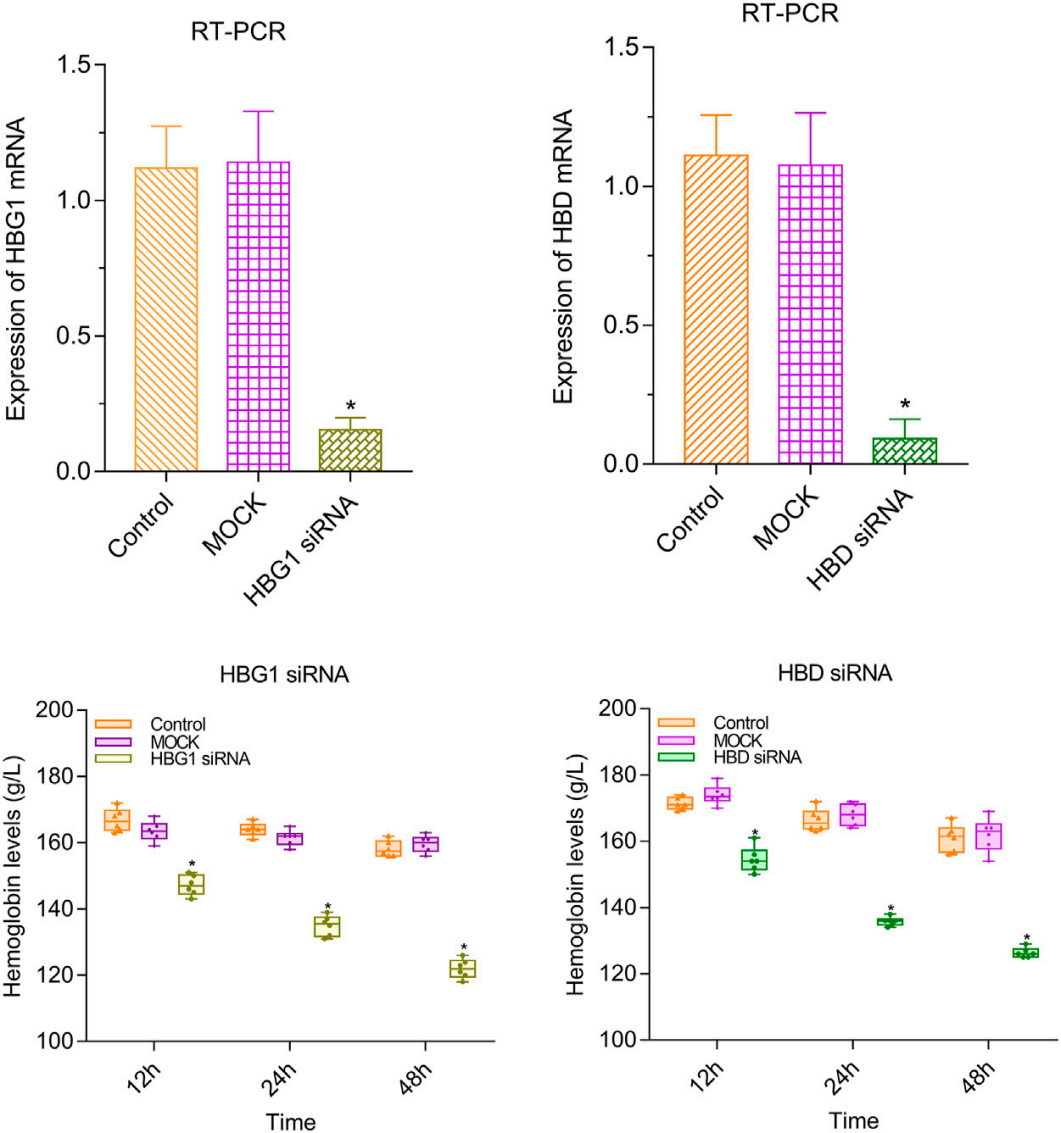
Lower level of hemoglobin in the red blood cells with HBG1-siRNA and HBG1-siRNA than the CON red blood cells.

Compared with the control and MOCK groups, the expressions of HBG1 and HBD were significantly downregulated in the red blood cells with HBG1-siRNA and HBG1-siRNA (*p* < 0.05). Furthermore, at every point in time, the level of hemoglobin was lower in the red blood cells with HBG1-siRNA and HBG1-siRNA significantly (Lim et al., 2020) (*p* < 0.05). With the passage of time (12, 24, and 48 h), the hemoglobin levels are getting lower and lower (*p* < 0.05; Figure 2).

### Better Effect of Edoxaban on the Pathogeny Structure of Cardiac Muscle Tissue

**FIGURE 3:**
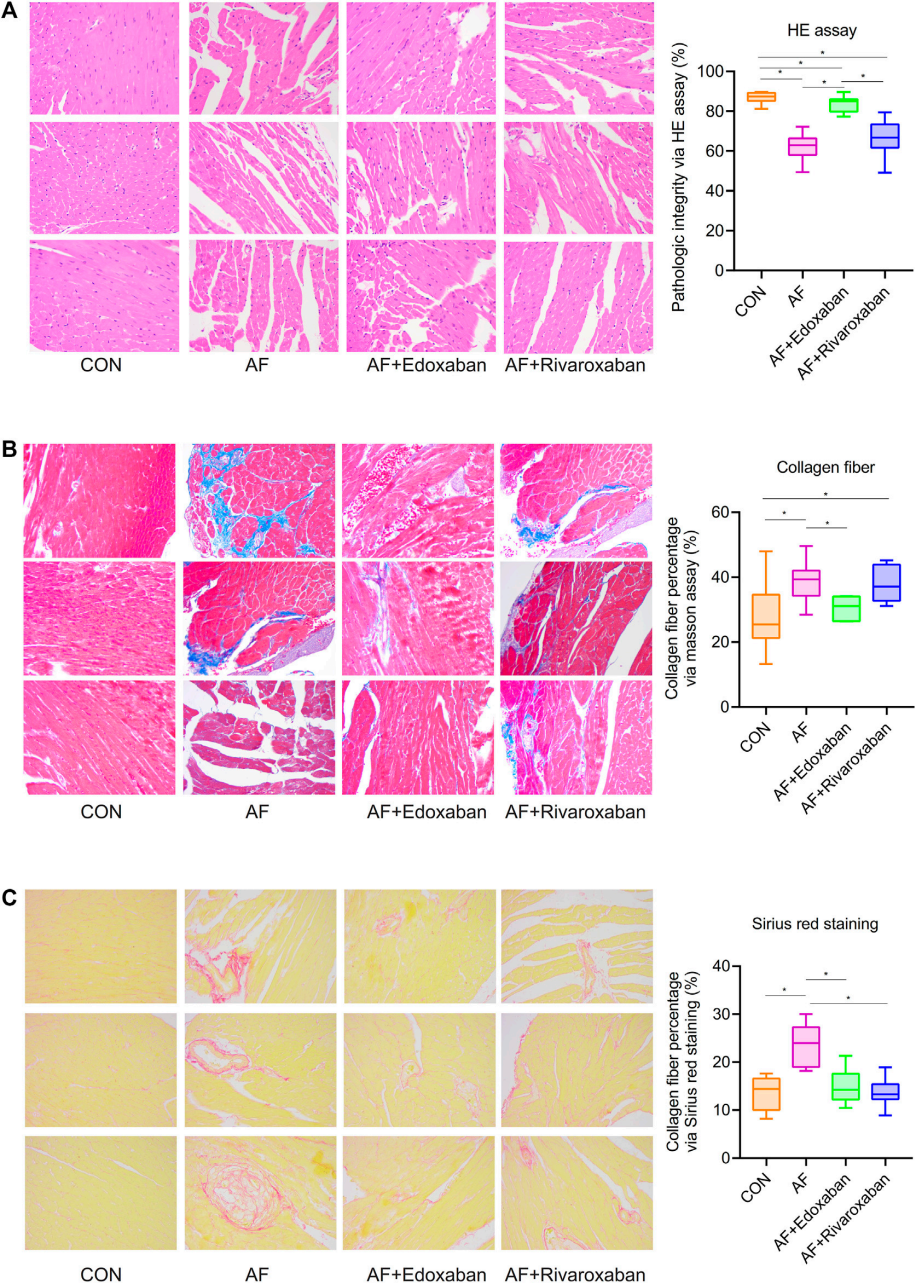
Better effect of edoxaban on the pathogeny structure of cardiac muscle tissue and the different degrees of myocardial fibrosis in the four groups. **(A)** Structure of cardiac muscle tissue in the AF + edoxaban group was better than in the AF group and the AF + rivaroxaban group. **(B)** Through the Masson assay, compared with the AF group, the collagen fiber percentage in the AF + edoxaban group was decreased significantly. **(C)** Through Sirius red staining, compared with the AF group, the collagen fiber percentage in the AF + edoxaban group was decreased significantly.

Compared with the control group, the structure of cardiac muscle tissue in the atrial fibrillation mice was destroyed more seriously (*p* < 0.05). However, the structure of cardiac muscle tissue in the AF + edoxaban group was better than in the AF group and the AF + rivaroxaban group (*p* < 0.05; Figure 3A).

### Different Degrees of Myocardial Fibrosis in the Four Groups

Through the Masson assay, the collagen fiber percentage in the AF group was significantly higher than in the CON group (*p* < 0.05), which manifested that myocardial fibrosis would be an occurrence in the AF cases (Gyöngyösi et al., 2017). Furthermore, compared with the AF group, the collagen fiber percentage in the AF + edoxaban group was decreased significantly (*p* < 0.05), which demonstrated that edoxaban is beneficial to the myocardium and reduced the situation of myocardial fibrosis (Figure 3B).

Furthermore, through the Sirius red staining, the aforementioned result by the Masson assay was demonstrated again. The collagen fibers are red in color. The collagen fiber percentage in the AF group was significantly higher than in the CON group (*p* < 0.05). Also, compared with the AF group, the collagen fiber percentage in the AF + edoxaban group was decreased significantly (*p* < 0.05; Figure 3C).

### Lower Degree of Destruction of Reticular Fibers in the Atrial Fibrillation + Edoxaban Group

**FIGURE 4:**
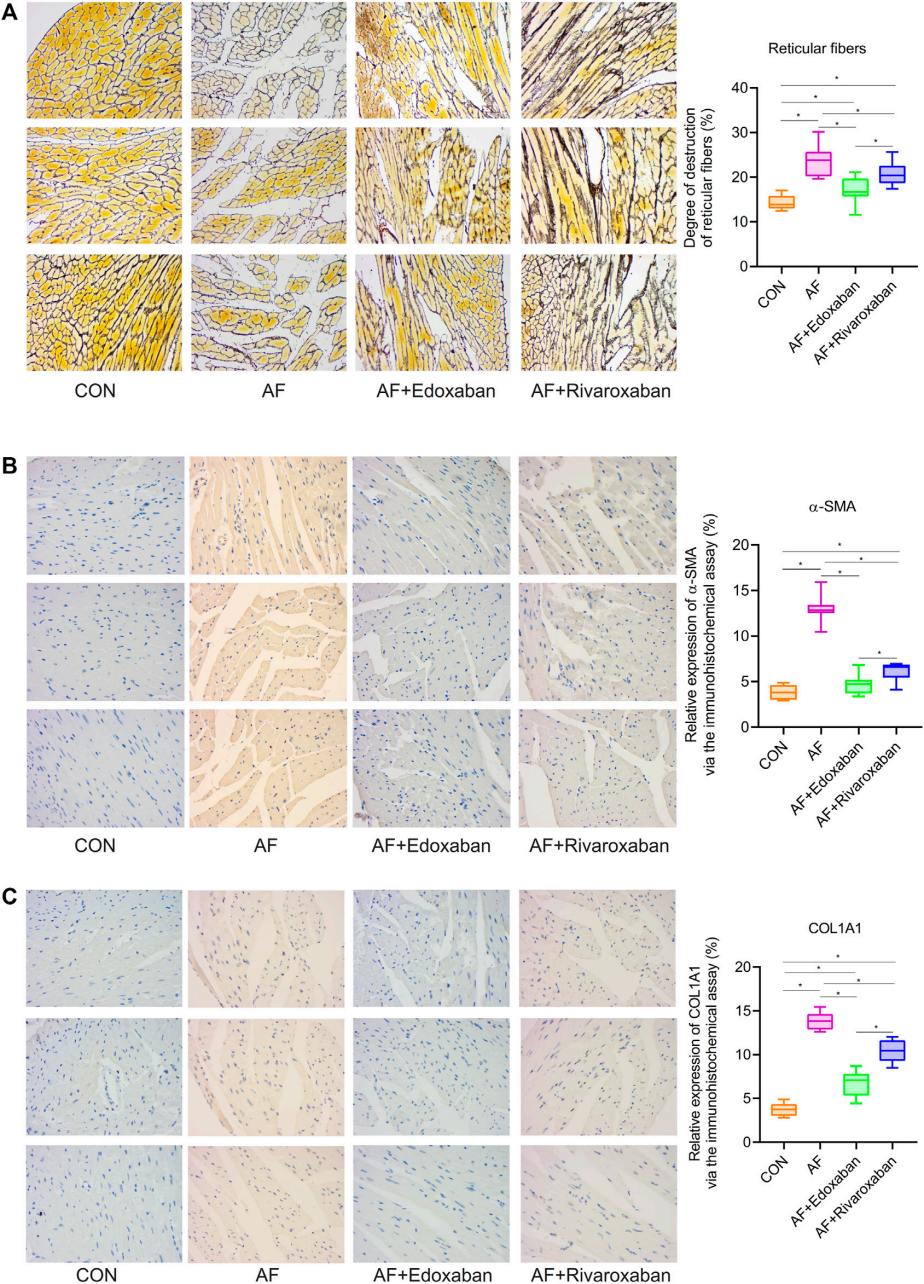
Lower degree of destruction of reticular fibers in the AF + edoxaban group and expressions of α-SMA and COL1A1 in the AF mice. **(A)** Degree of destruction of reticular fibers in the AF + edoxaban group or the AF + rivaroxaban group was lower than the AF group. After using edoxaban or rivaroxaban, the expressions of **(B)** α-SMA and **(C)** COL1A1 were significantly decreased *via* the immunohistochemistry assay (*p* < 0.05).

Through the reticular fiber staining, compared with the CON group, the degree of destruction of reticular fibers was significantly increased in the AF group (*p* < 0.05). However, the degree of destruction of reticular fibers in the AF + edoxaban group or the AF + rivaroxaban group was lower than that in the AF group. In addition, the degree of destruction of reticular fibers was higher in the AF + rivaroxaban group than in the AF + edoxaban group (*p* < 0.05). (Figure 4A).

### Expressions of α-SMA and COL1A1 in the Atrial Fibrillation Mice

By the immunohistochemistry assay, the expressions of α-SMA and COL1A1 in the cardiac muscle tissue in the AF group were upregulated compared with the CON group (*p* < 0.05). After using edoxaban or rivaroxaban, the expressions of α-SMA and COL1A1 were significantly decreased (*p* < 0.05). Furthermore, compared with the AF + rivaroxaban group, the expressions of α-SMA and COL1A1 in the AF + edoxaban group were downregulated (*p* < 0.05) (Figure 4B,C).

**FIGURE 5:**
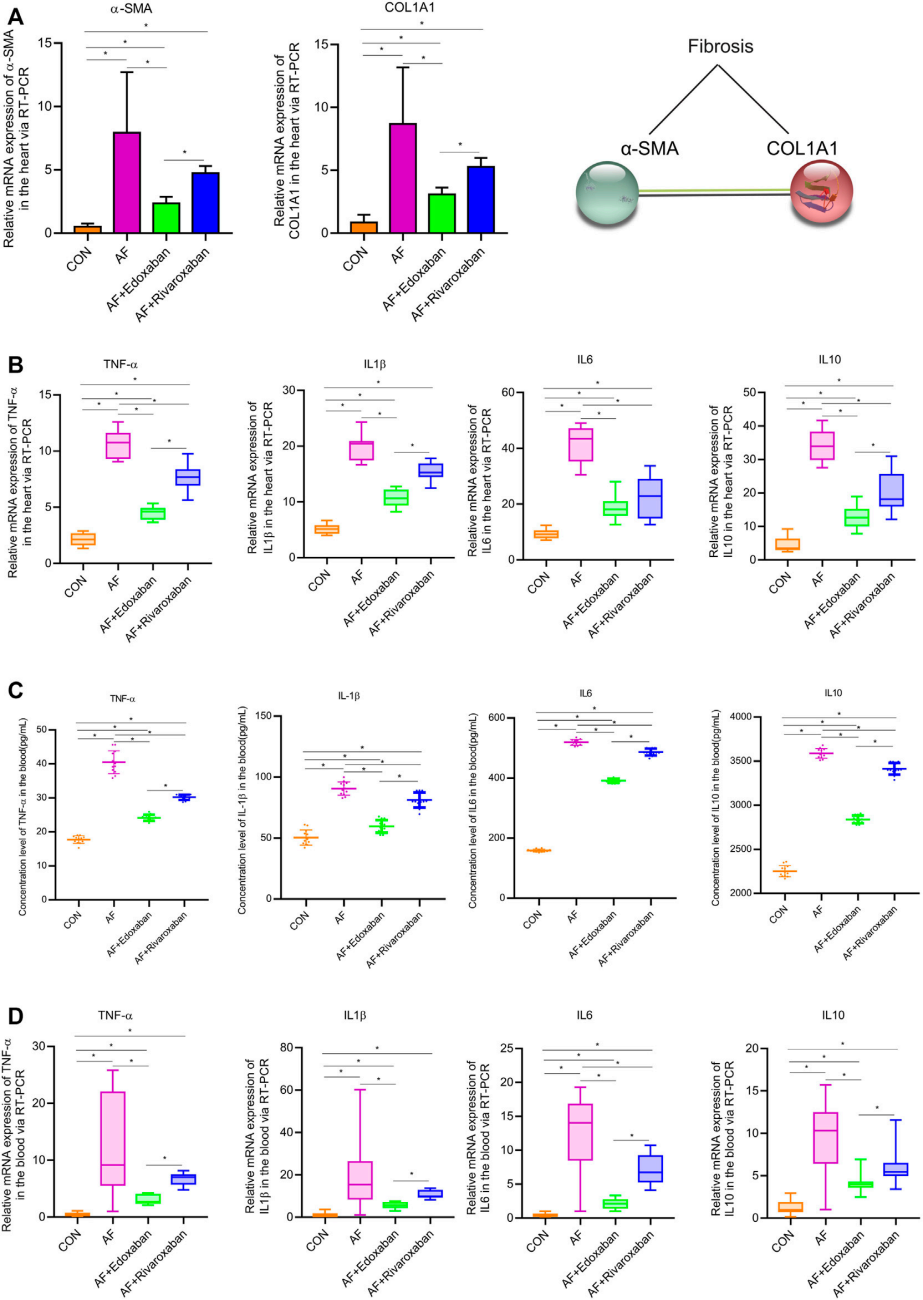
mRNA levels of α-SMA and COL1A1 via the RT-PCR, and edoxaban reduced the level of inflammation in the heart of the AF mouse model. **(A)** Compared with the AF group, the mRNA expressions of α-SMA and COL1A1 in the cardiac muscle tissue were downregulated in the AF + edoxaban group and AF + rivaroxaban group (*p* < 0.05). **(B)** After using edoxaban or rivaroxaban, the mRNA expressions of inflammatory biomarkers (TNF-α, IL-1β, IL-6, and IL-10) in the heart were significantly decreased *via* the RT-PCR (*p* < 0.05). **(C)** Through the ELISA, the inflammatory biomarkers (TNF-α, IL-1β, IL-6, and IL-10) in the blood were upregulated in the AF group than in the AF + edoxaban group. **(D)** Inflammatory biomarkers (TNF-α, IL-1β, IL-6, and IL-10) in the blood were also detected via the RT-PCR assay. Compared with the AF group, the mRNA expressions of TNF-α, IL-1β, IL-6, and IL-10 were downregulated in the AF + edoxaban group and the AF + rivaroxaban group (*p* < 0.05).

In addition, the aforementioned results were demonstrated via the RT-PCR. At the mRNA level, the expressions of α-SMA and COL1A1 in the cardiac muscle tissue in the AF group were upregulated compared with the CON group (*p* < 0.05). Compared with the AF group, the expressions of α-SMA and COL1A1 in the cardiac muscle tissue were downregulated in the AF + edoxaban group and the AF + rivaroxaban group (*p* < 0.05). Furthermore, there exists a significant interaction between the α-SMA and COL1A1, which were all the parameters reflecting the degree of fibrosis. (Figure 5A).

### Edoxaban Reduced the Level of Inflammation in the Heart of the Atrial Fibrillation Mouse Model

The inflammatory biomarkers (TNF-α, IL-1β, IL-6, and IL-10) in the heart were detected via the RT-PCR assay. Compared with the CON group, the expressions of inflammatory biomarkers (TNF-α, IL-1β, IL-6, and IL-10) in the AF group were higher. After using edoxaban or rivaroxaban, the expressions of inflammatory biomarkers (TNF-α, IL-1β, IL-6, and IL-10) were significantly decreased (*p* < 0.05). Furthermore, compared with the AF + rivaroxaban group, the expressions of inflammatory biomarkers (TNF-α, IL-1β, IL-6, and IL-10) in the AF + edoxaban group were downregulated (*p* < 0.05), which presented that edoxaban was superior to rivaroxaban in the aspect of reducing the inflammation in the AF mice (Figure 5B).

### Lower Expression of the Inflammatory Biomarkers in the Blood of Atrial Fibrillation + Edoxaban Mice

Through the ELISA, the inflammatory biomarkers (TNF-α, IL-1β, IL-6, and IL-10) in the blood were upregulated in the AF group than in the CON group (*p* < 0.05). However, compared with the AF group, the expressions of inflammatory biomarkers (TNF-α, IL-1β, IL-6, and IL-10) were downregulated in the AF + edoxaban group and the AF + rivaroxaban group (*p* < 0.05). Furthermore, the expressions of inflammatory biomarkers in the AF + edoxaban group were lower than in the AF + rivaroxaban group (Figure 5C). The inflammatory biomarkers (TNF-α, IL-1β, IL-6, and IL-10) in the blood were also detected via the RT-PCR assay, and the results were further demonstrated (Figure 5D).

### Effect of Edoxaban on Decreasing Blood Lipids in the Atrial Fibrillation Mice

**FIGURE 6:**
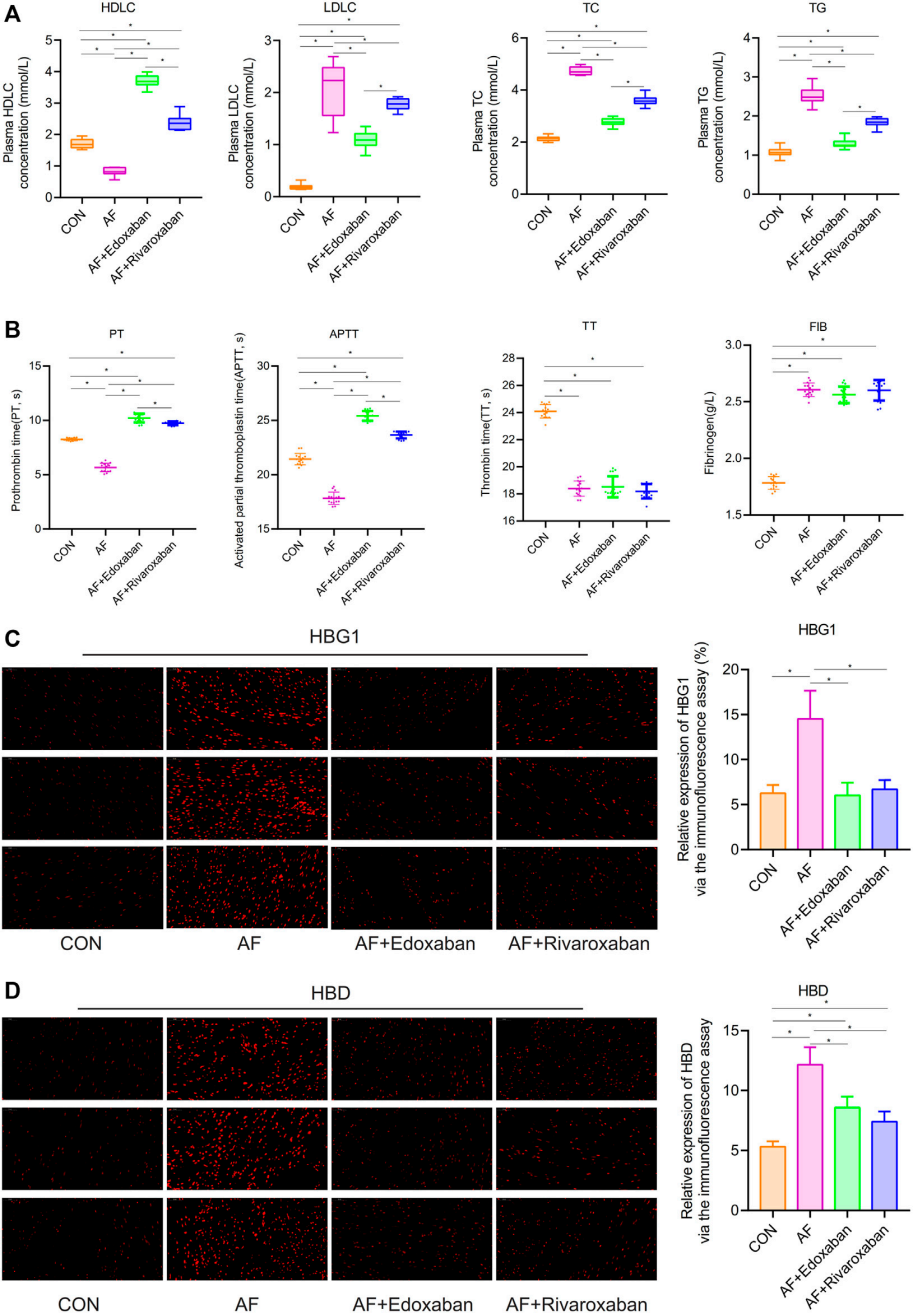
Effect of edoxaban on decreasing blood lipids, coagulation, HBG1, and HBD in the AF mice. **(A)** Compared with the AF group, the blood lipids’ parameters (LDL-C, TC, and TG) were downregulated in the AF + edoxaban group and the AF + rivaroxaban group. **(B)** PT and APTT in the AF + edoxaban group and the AF + rivaroxaban group were more increasing than in the AF mice. **(C,D)** Compared with the AF group, the expressions of **(C)** HBG1 and **(D)** HBD were downregulated in the AF + edoxaban group and the AF + rivaroxaban group (*p* < 0.05) through the immunofluorescence assay.

Compared with the CON group, plasma HDL-C concentration in the AF group was significantly lower (*p* < 0.05). However, the plasma HDL-C concentration in the AF + edoxaban group and AF + rivaroxaban group was higher than in the AF mice (*p* < 0.05). In addition, compared with the AF + rivaroxaban group, the plasma HDL-C concentration in the AF + edoxaban group was higher (*p* < 0.05). The blood lipids’ parameters (LDL-C, TC, and TG) were upregulated in the AF group compared with the CON group (*p* < 0.05). However, compared with the AF group, the blood lipids’ parameters (LDL-C, TC, and TG) were downregulated in the AF + edoxaban group and the AF + rivaroxaban group (*p* < 0.05). Furthermore, the blood lipids’ parameters (LDL-C, TC, and TG) in the AF + edoxaban group were lower than in the AF + Rivaroxaban group (Figure 6A).

### Reducing Coagulation by the Edoxaban in the Atrial Fibrillation Mice

Compared with the CON group, PT and APTT in the AF group were significantly decreasing (*p* < 0.05). However, the PT and APTT in the AF + edoxaban group and AF + rivaroxaban group were more increasing than in the AF mice (*p* < 0.05). In addition, compared with the AF + rivaroxaban group, the PT and APTT in the AF + edoxaban group were longer (*p* < 0.05). Compared with the CON group, the TT was significantly shorter in the AF, the AF + edoxaban, and the AF + rivaroxaban groups (*p* < 0.05). However, there were no differences among the three groups (AF, AF + edoxaban, and AF + rivaroxaban group) in the aspect of TT. Compared with the CON group, the FIB was significantly higher in the AF, the AF + edoxaban, and the AF + rivaroxaban groups (*p* < 0.05). However, there were no differences among the three groups (AF, AF + edoxaban, and AF + rivaroxaban groups) in the aspect of FIB (Figure 6B).

### Effect of Edoxaban in Reducing HBG1 and HBD in the Atrial Fibrillation Mouse Model

**FIGURE 7:**
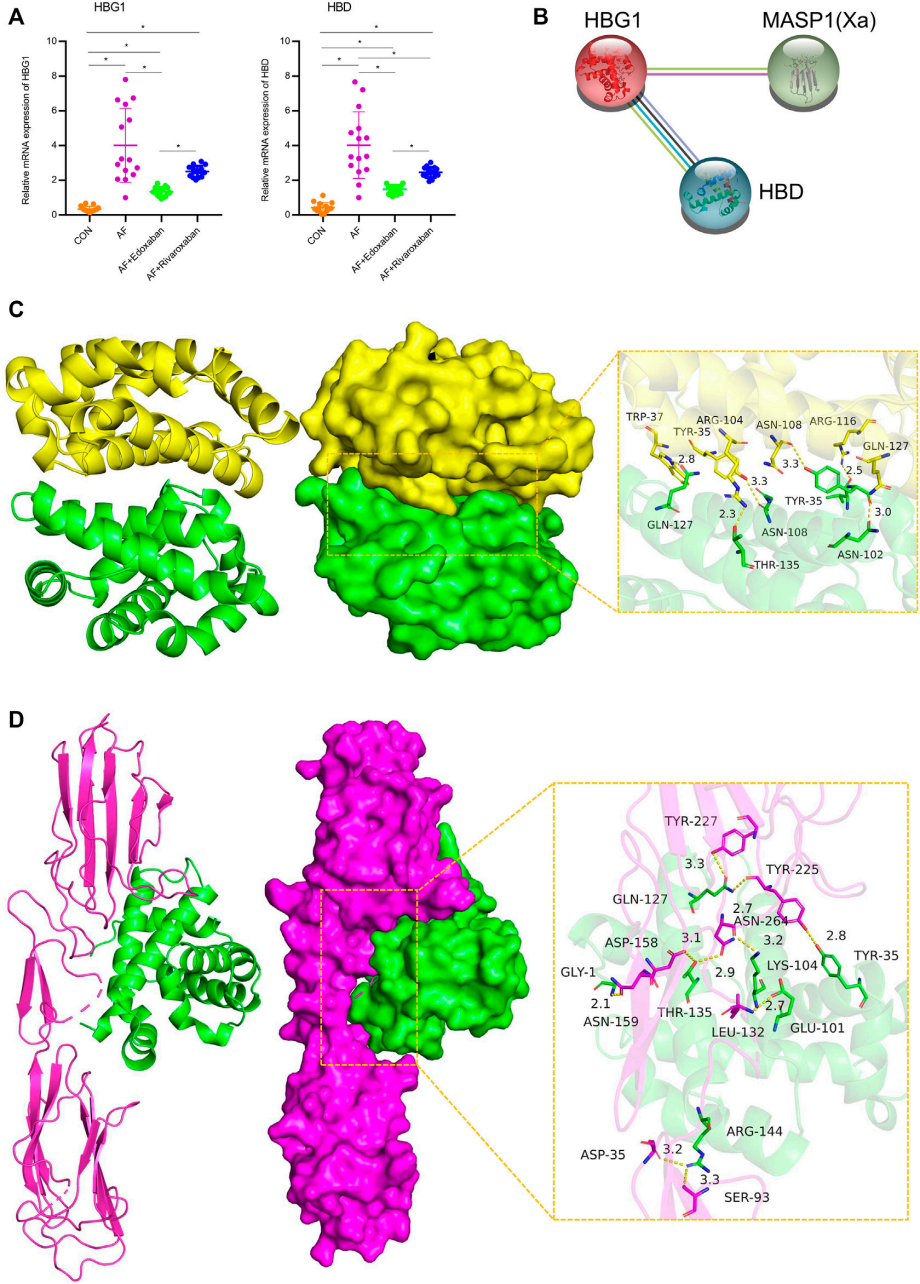
mRNA expressions of HBG1 and HBD via the RT-PCR and the interaction between HBG1, HBD, and MASP1 (Xa). **(A)** In the PCR assay, the results showed that the expressions of HBG1 and HBD in the AF + edoxaban group were lower than in the AF + rivaroxaban group. **(B)** There exists a significant interaction between HBG1, HBD, and MASP1(Xa). **(C)** Binding mode of the complex HBG1 with HBD. The backbone of protein was rendered in tube and colored in green (HBG1) and yellow (HBD). HBG1 and HBD proteins are rendered by the surface. The detail binding mode of HBG1 with HBD. Yellow dash represents the hydrogen bond or salt bridge. **(D)** Binding mode of the complex HBG1 with MASP1. The backbone of protein was rendered in tube and colored in green (HBG1) and red (MASP1). HBG1 and MASP1 proteins are rendered by the surface. The detailed binding mode of HBG1 with MASP1. Yellow dash represents the hydrogen bond or salt bridge.

Through immunofluorescence, the expressions of HBG1 and HBD were upregulated in the AF group compared with those of the CON group (*p* < 0.05). However, compared with the AF group, the expressions of HBG1 and HBD were downregulated in the AF + edoxaban group and the AF + rivaroxaban group (*p* < 0.05) (Figure 6C). In addition, the results were also demonstrated by the RT-PCR assay. Furthermore, in the PCR assay, the results showed that the expressions of HBG1 and HBD in the AF + edoxaban group were lower than in the AF + rivaroxaban group (Figure 7A). Also, there exists a significant interaction between HBG1, HBD, and MASP1(Xa), which were the parameters reflecting the coagulation function (Figure 7B).

### Molecular Docking Among HBG1, HBD, and MASP1(Xa)

The binding score of HBG1 to HBD protein was −240.26 kcal/mol. The binding site of HBG1 protein included THR-135, GLN-127, ASN-108, TYR-35, VAL-33, and ASN-102. The binding site of HBD protein included GLN-127, ARG-116, ASN-108, TYR-35, TRP-37, and ARG-104. HBG1 and HBD protein contact residues can form a variety of interactions, such as salt bridge, hydrogen bond, hydrophobic interaction, and other interactions. Also, these interactions can effectively improve the stability of the HBG1 and HBD protein complex. In addition, according to the binding surface diagram of the two proteins, it was found that HBD protein matched well with HBG1 protein surface, which was conducive to forming a stable binding effect (Figure 7C; Table 2).

**TABLE 2:** Docking results of three target proteins.

Protein 1	Protein 2	Binding energy (kcal/mol)	Contact sites (protein 1)	Contact sites (protein 2)	Combination type
HBG1	HBD	−240.26	THR-135, GLN-127, ASN-108, TYR-35, VAL-33, and ASN-102	GLN-127, ARG-116, ASN-108, TYR-35, TRP-37, and ARG-104	Salt bridge, hydrogen bond, and hydrophobic interaction
HBG1	MASP1	−256.39	ARG-144, TYR-35, GLN-127, THR-135, LYS-104, GLU-101, and GLY-1	ASN-159, LEU-132, ASN-264, ASP-158, SER-93, ASP-35, TYR-225, and TYR-227	Salt bridge, hydrogen bond, and hydrophobic interaction

The binding score of HBG1 to MASP1 protein was −256.39 kcal/mol. The binding site of HBG1 protein included ARG-144, TYR-35, GLN-127, THR-135, LYS-104, GLU-101, ad GLY-1. The binding site of MASP1 protein included ASN-159, LEU-132, ASN-264, ASP-158, SER-93, ASP-35, TYR-225, and TYR-227. HBG1 and MASP1 protein contact residues can form a variety of interactions, such as salt bridge, hydrogen bond, hydrophobic interaction, and other interactions. Also, these interactions can effectively improve the stability of HBG1 and MASP1 protein complex. In addition, according to the binding surface diagram of the two proteins, it was found that MASP1 protein matched well with HBG1 protein surface, which was conducive to forming a stable binding effect (Figure 7D; Table 2).

### Successful Construction of the BP Neural Network Among HBG1, HBD, and PT

**FIGURE 8:**
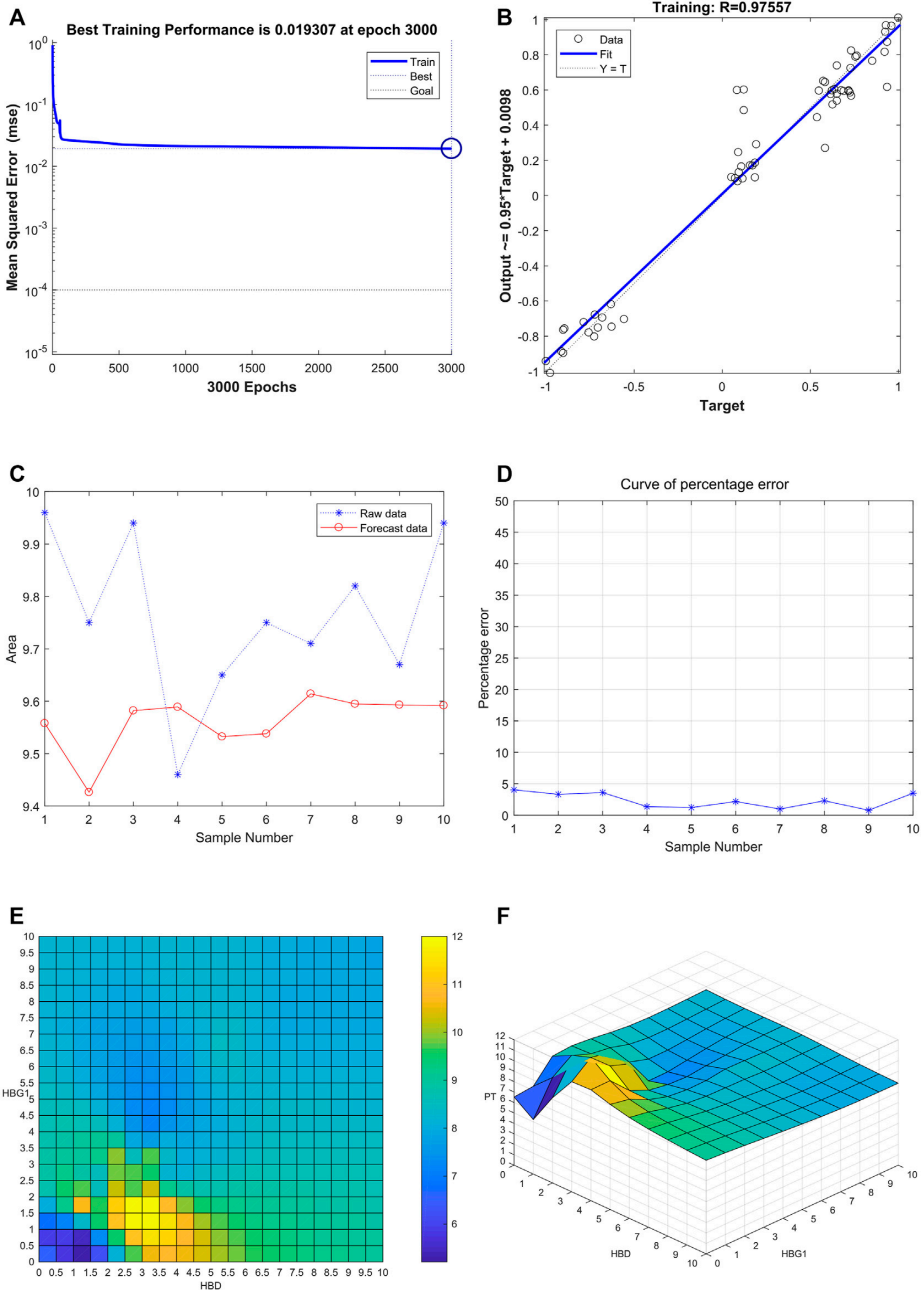
Successful construction of the BP neural network among HBG1, HBD and PT. **(A)** After training of 3000 epochs, the best training performance is 0.019307. **(B)** There exists a significant interaction between HBG1, HBD, and PT. Also, the correlation coefficient is 0.97557. **(C)** Variation tendency between the raw data and forecast data was close. **(D)** Curve of the percentage error was also drawn, and the percentage errors were all less than the 5%. **(E,F)** Quantitative predictions of HBG1 and HBD for the PT in the cubic spline interpolation were performed, and when 2.0 < HBD<5.0, 0 < HBG1<3.5, the value of PT is larger.

After training of 3000 epochs, the best training performance is 0.019307, which is less than 0.05, showing that it is of significance (Figure 8A). The correlation (R), calculated from the network, is 0.97557. Also, there exists a significant interaction among HBG1, HBD, and PT (Figure 8B). Furthermore, the model was verified by the ten individuals, and the variation tendency between the raw data and forecast data was close (Figure 8C). The curve of percentage error was also drawn, and the percentage errors were all less than 5% (Figure 8D). Quantitative predictions of HBG1 and HBD for the PT in the cubic spline interpolation were performed, and when 2.0 < HBD<5.0, 0 < HBG1<3.5, the value of PT is larger (Figure 8E,F).

### Significant Effect of HBG1 and HBD on the Blood Coagulation Function Based on the Support Vector Machine

**FIGURE 9:**
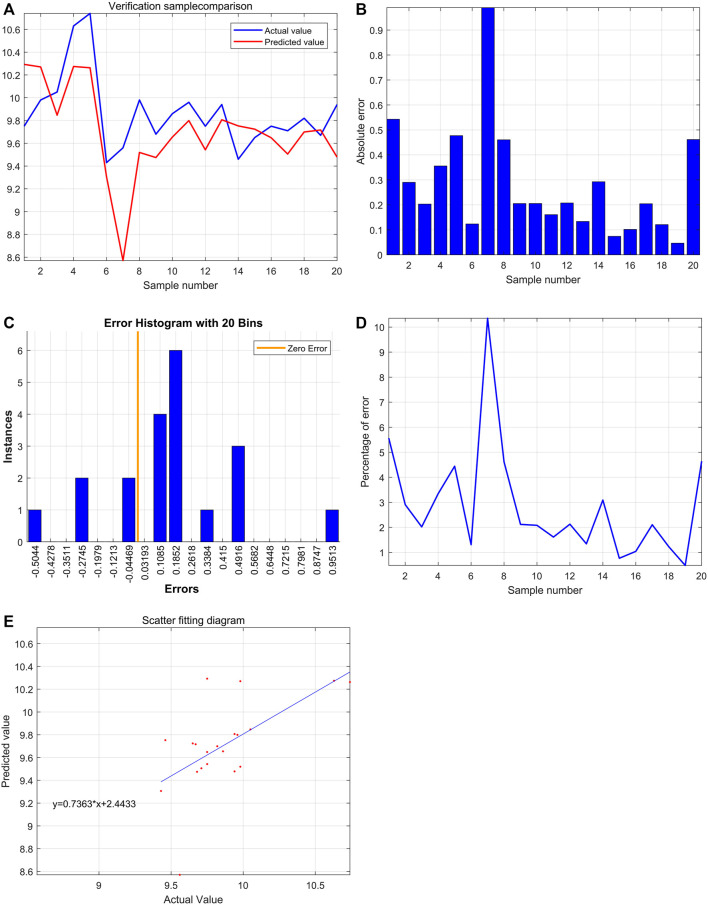
Significant effect of HBG1 and HBD on the blood coagulation function based on the support vector machine. **(A)** Through the comparison between actual value and predicted value, the variation tendency was similar. **(B)** Most absolute errors were less than 0.5. **(C)** Error histogram with 20 bins. **(D)** Curve of the percentage error was also drawn, and the most percentage errors were all less than 5%. **(E)** In the scatter fitting diagram, the relationship between the predicted value and the actual value is given by y = 0.7363*X+2.4433.

Through the comparison between the actual value and the predicted value, the variation tendency was similar. The model that used HBG1 and HBD expressions to predict PT was valuable (Figure 9A). Then, the most absolute errors were less than 0.5 (Figure 9B). The error histogram with 20 bins is shown in Figure 9C. The curve of percentage error was also drawn, and the most percentage errors were all less than 5% (Figure 9D). In the scatter fitting diagram, the relationship between the predicted value and the actual value is given by y = 0.7363*X+2.4433 (Figure 9E).

### A Strong Interaction Between HBG1 and HBD

**FIGURE 10:**
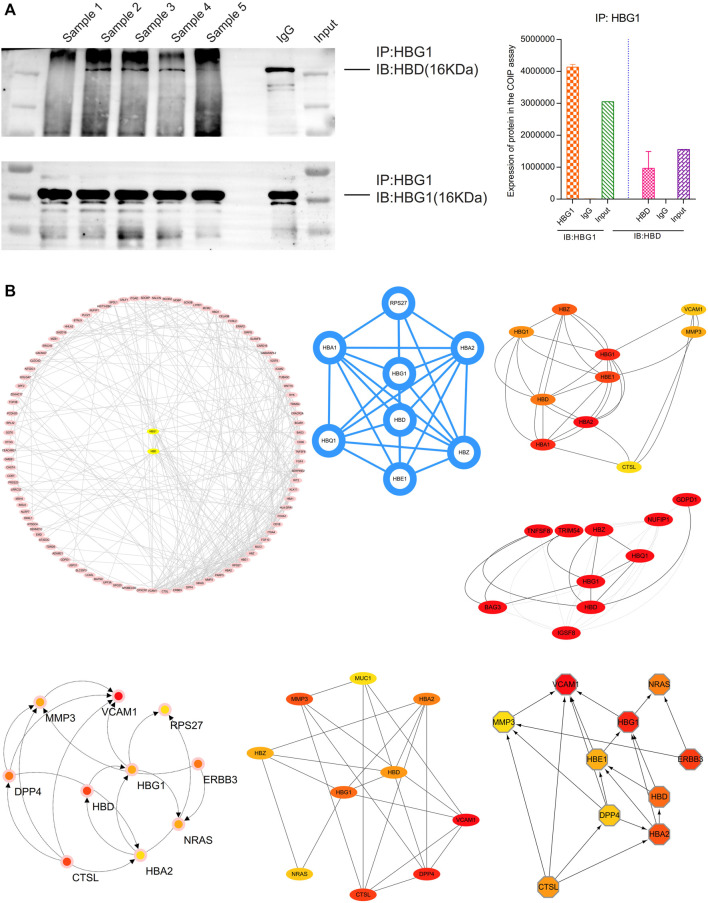
Strong interaction between HBG1 and HBD. **(A)** HBG1 and HBD were co-expressed in the blood via the COIP assay. **(B)** HBG1 and HBD were at the core of the PPI network. Furthermore, through calculation by MCODE and cytoHubba, HBG1 and HBD were hub genes in the network, and there was a strong interaction between HBG1 and HBD.

HBG1 and HBD were co-expressed in the blood via the COIP assay, which showed that when HBG1 occurred in the samples, the HBD was also expressed (Figure 10A). The protein–protein interaction (PPI) network presented a strong interaction between the genes related to HBG1 and HBD. Furthermore, HBG1 and HBD were at the core of the PPI network. Furthermore, through calculation by the MCODE and cytoHubba, HBG1 and HBD were hub genes in the network, and there was a strong interaction between HBG1 and HBD (Figure 10B).

### Enrichment Analysis for the Relative Genes of HBG1 and HBD

**FIGURE 11:**
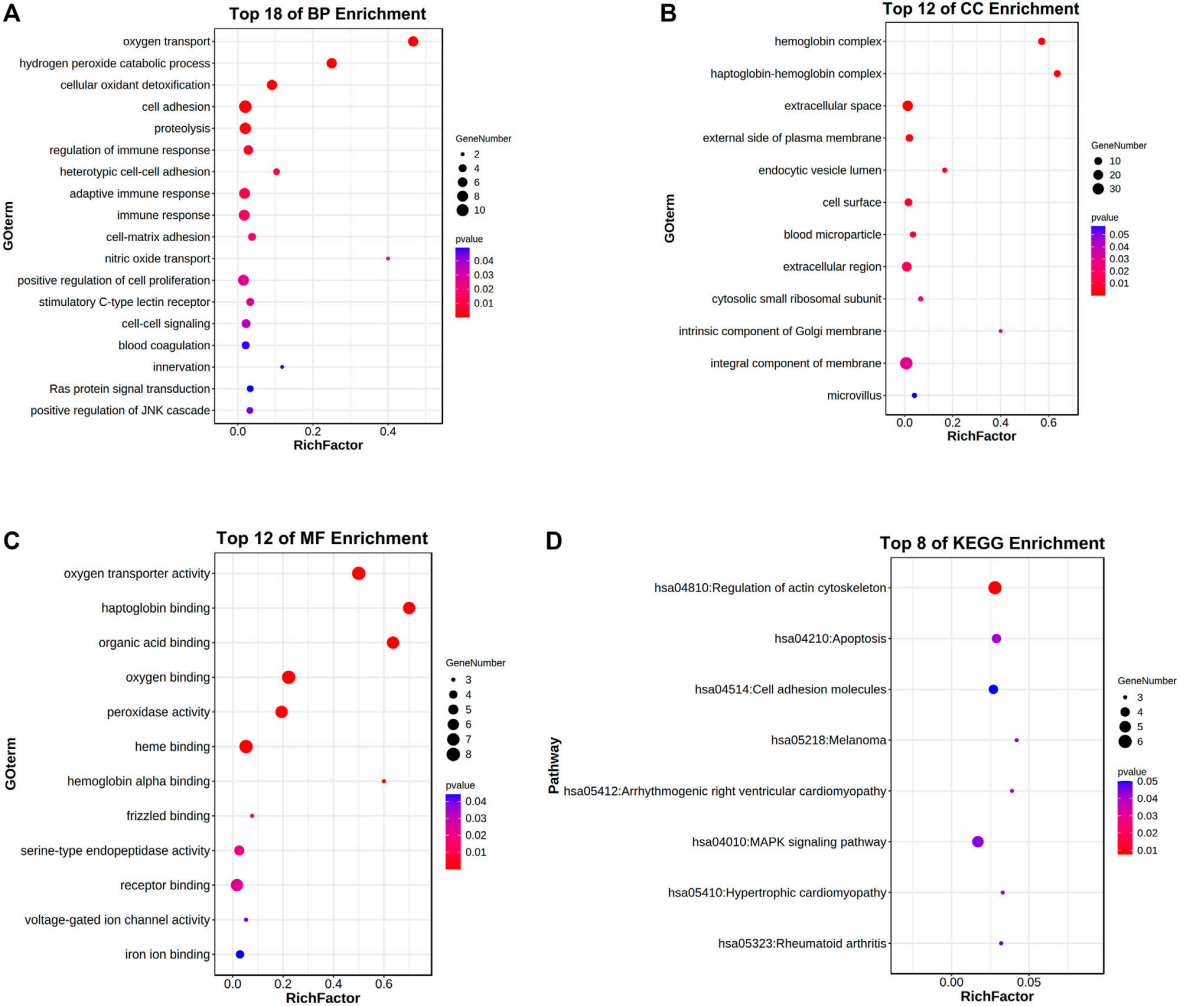
Enrichment analysis for the relative genes of HBG1 and HBD via the DAVID. **(A)** In the aspect of the biological process (BP), the relative genes of HBG1 and HBD were mainly enriched in blood coagulation and in the regulation of immune response. **(B)** In the aspect of the cellular component (CC), the relative genes of HBG1 and HBD were mainly enriched in the blood microparticle, extracellular region, haptoglobin–hemoglobin complex, and hemoglobin complex. **(C)** In the aspect of cellular molecular function (MF), the relative genes of HBG1 and HBD were mainly enriched in the hemoglobin alpha binding, oxygen binding, oxygen transporter activity, and haptoglobin binding. **(D)** In the aspect of KEGG, the relative genes of HBG1 and HBD were mainly enriched in apoptosis, cell adhesion molecules, and regulation of actin cytoskeleton.

Through the DAVID analysis, in the aspect of the biological process (BP), the relative genes of HBG1 and HBD were mainly enriched in the blood coagulation, regulation of immune response, positive regulation of JNK cascade, and stimulatory C-type lectin receptor. In the BP analysis, the *p*-values of terms in the top 18 were less than 0.05 (Figure 11A). In the aspect of the cellular component (CC), the relative genes of HBG1 and HBD were mainly enriched in the blood microparticle, extracellular region, haptoglobin–hemoglobin complex, and hemoglobin complex (Figure 11B). In the aspect of cellular molecular function (MF), the relative genes of HBG1 and HBD were mainly enriched in hemoglobin alpha binding, oxygen binding, oxygen transporter activity, and haptoglobin binding. The top 12 in CC and MF were less than 0.05, but others were more than 0.05. (Figure 11C). In the aspect of KEGG, the relative genes of HBG1 and HBD were mainly enriched in apoptosis, cell adhesion molecules, and regulation of actin cytoskeleton (Figure 11D).

**FIGURE 12:**
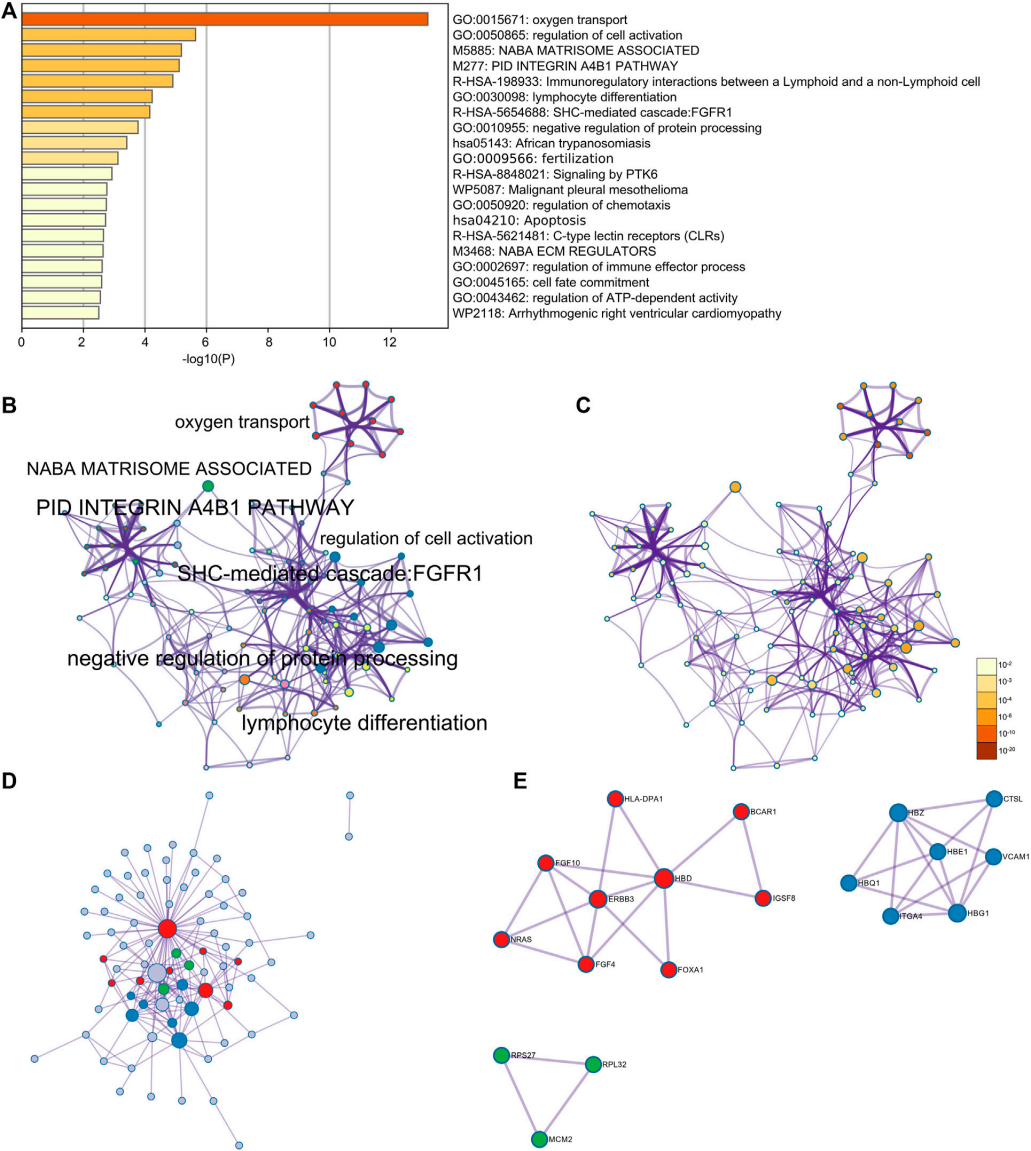
Enrichment analysis for the relative genes of HBG1 and HBD *via* Metascape. **(A)** Relative genes of HBG1 and HBD were mainly enriched in oxygen transport, regulation of cell activation, apoptosis, and regulation of the ATP-dependent activity. **(B)** Network of enriched terms colored by cluster ID. **(C)** Network of enriched terms colored by the *p*-value. **(D,E)** Enrichment PPI network by the Metascape and MCODE components identified in the gene lists showed that HBG1 and HBD were at the core of the network.

Furthermore, through the Metascape analysis, the relative genes of HBG1 and HBD were mainly enriched in oxygen transport, regulation of cell activation, apoptosis, and regulation of ATP-dependent activity (Figure 12A). Figure 12B shows the network of enriched terms colored by cluster ID, where nodes that share the same cluster ID are typically close to each other, and Figure 12C presents the network of enriched terms colored by *p*-value (*p* < 0.05). The enrichment PPI network by the Metascape and MCODE components identified in the gene lists showed that HBG1 and HBD were at the core of the network (Figure 12D,E).

### Small Molecule Compounds Related to HBG1 and HBD

**FIGURE 13:**
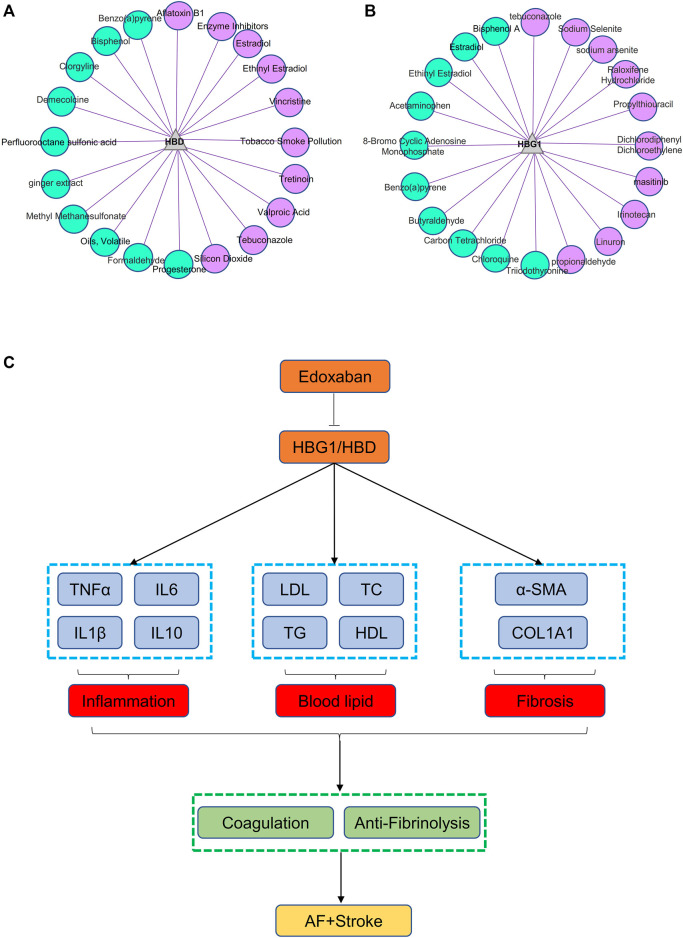
Small molecule compounds related to HBG1 and HBD and the general idea diagram of the effect of edoxaban on the AF. **(A)** Small molecule compounds related to HBD. **(B)** Small molecule compounds related to HBG1. **(C)** General idea diagram of the effect of edoxaban on the AF. Edoxaban might inhibit the expressions of HBG1 and HBD and then inhibit inflammation, blood lipids, and fibrosis further. Furthermore, coagulation was inhibited, and fibrinolysis was activated at the effect of edoxaban. Finally, the development of AF to the stroke was prevented.

HBD might be involved in the metabolic process of small molecule compounds including enzyme inhibitors, estradiol, ethinylestradiol, and vincristine, which might be related to edoxaban (Figure 13A). HBG1 might be involved in the metabolic process of small molecule compounds including tebuconazole, sodium selenite, sodium arsenite, propylthiouracil, and propionaldehyde, which might be related to edoxaban (Figure 13B). Through CTD analysis, we found that the small molecule compounds involved in HBG1/HBD contain an important component of edoxaban (methyl methanesulfonate), and then concluded that edoxaban can effectively reduce lipids and fibrosis through HBG1/HBD biomarkers to prevent AF and coagulation.

### General Idea Diagram of the Effect of Edoxaban on the Atrial Fibrillation

Edoxaban might inhibit the expression of HBG1 and HBD and then inhibit the inflammation, blood lipids, and fibrosis further. Furthermore, the coagulation was inhibited and the fibrinolysis was activated at the effect of edoxaban. Finally, the development of AF to the stroke was prevented (Figure 13C).

## Discussion

Atrial fibrillation (AF) is the most common persistent arrhythmia in adults (Hindricks et al., 2021). Uncoordinated atrial excitation can lead to the deterioration of cardiac function (Subahi et al., 2018), and the occurrence of atrial fibrillation can also lead to the formation of atrial mural thrombus, which in turn leads to thromboembolic events such as ischemic stroke, myocardial infarction, and peripheral arterial embolism (Tsuchiya et al., 1992; Hossmann 2006; Pandya et al., 2011). Atrial fibrillation can double the risk of ischemic stroke, and stroke caused by atrial fibrillation often has a poor prognosis. Also, stroke has also become one of the important causes of death in patients with atrial fibrillation. Therefore, the prevention of thromboembolic events caused by atrial fibrillation is the cornerstone of the treatment of atrial fibrillation. Our results showed that edoxaban might inhibit the expression of HBG1 and HBD and then inhibit inflammation, blood lipids, and fibrosis further. The expressions of HBG1 and HBD in the red blood cells of the patients with atrial fibrillation were decreased. Compared with the AF group, the expressions of HBG1 and HBD were downregulated in the AF + edoxaban group (*p* < 0.05).

The results showed that after using edoxaban or rivaroxaban, the expressions of inflammatory biomarkers (TNF-α, IL-1β, IL-6, and IL-10) were significantly decreased (*p* < 0.05). Atrial fibrillation (AF) is the most common clinical arrhythmia. There is strong evidence for a systemic prothrombotic and proinflammatory state in AF. Inflammation is an important link in the pathophysiology of cerebrovascular diseases, especially ischemic stroke. A study showed that post-stroke neuroinflammation is an important factor affecting the long-term prognosis of ischemia. Various factors, such as ROS formation, necrotic cells, and damaged tissue, can cause the activation of inflammatory cells, which can cause an inflammatory response. Furthermore, these activated inflammatory cytokines are involved in tissue remodeling. The inflammatory process is associated with several different cell types, inflammatory cytokines, and cellular receptors such as Toll-like receptors (TLRs) (Fernandes et al., 2014).

There is increasing evidence that FXa exerts non-hemostatic cellular effects, is mediated primarily through protease-activated receptor 1, and is associated with pathophysiological conditions such as atherosclerosis, inflammation, and fibrosis. FXa induces the expression of proinflammatory cytokines, including interleukin-6 (IL-6) and IL-8 and monocyte chemoattractant protein-1 (MCP-1) in ECs, promoting inflammatory processes (Busch et al., 2005; Daubie et al., 2006). FXa can exert a protective physiological role in endothelial cells but under specific conditions can lead to an inflammatory response by activating PAR-1, PAR-2, and possibly PAR-3 if FXa is activated uncontrollably, leading to an increased expression in inflammatory cytokines and adhesion molecules in ECs. Factor Xa is an important target for antithrombotic therapy due to its critical role in coagulation and thrombosis (Esmon 2014). In recent years, three DOACs (rivaroxaban, apixaban, and edoxaban) have been developed that inhibit the effects of FXa. Direct oral anticoagulants are small molecules that bind rapidly and reversibly to the active site of FXa, exhibiting higher selectivity compared with other serine proteases. FXa and atrial tachyarrhythmias act synergistically, resulting in an increased expression of myocardial PARs and subsequent activation of ERK/MAPK and NF-kb pathways. As a result, the expression of inflammatory molecules increases. Rivaroxaban inhibits the activation of ERK/MAPK and NF-kb and the expression of inflammatory molecules in human atrial tissue (32). Another study showed that rivaroxaban elicits anti-inflammatory end products (AGEs) by inhibiting the production of reactive oxygen species and the formation of advanced glycation, as well as the genes for MCP-1 and ICAM-1 in AGE-exposed HUVEC Express. Previous studies have shown that FXa inhibitors such as rivaroxaban have anti-inflammatory properties in addition to their anticoagulant effects (Bukowska et al., 2013; Katoh et al., 2017).

Edoxaban, the latest DOAC, is an oral factor Xa inhibitor. One study evaluated the effects of edoxaban on coagulation parameters, microvascular thrombosis, organ injury parameters, and inflammatory cytokines in endotoxin-injected rats. In this LPS-induced thrombosis model, the direct FXa inhibitor edoxaban significantly inhibited hypercoagulability, fibrin deposition in the liver, and elevation of liver injury parameters in a dose-dependent manner. Edoxaban significantly inhibited coagulation activation, intrahepatic microvascular thrombosis, and liver injury in endotoxin-injected rats, and reduced mortality. However, edoxaban did not cause kidney damage and inflammatory cytokine production. These results suggest that FXa inhibition by edoxaban may be a beneficial therapy for reducing inflammation-related thrombosis (Morishima et al., 2021).

The anti-inflammatory effects of FXa inhibitors were further demonstrated in a study in which rivaroxaban or apixaban treatment for 6 months reduced the pentaxin-related protein (PTX-3), a protein mainly produced by macrophages and vascular endothelial cells, in response to early proinflammatory signals. The authors then propose that PTX3 rapidly responds to left atrial and vascular endothelial changes as a useful marker for determining the anti-inflammatory effects of FXa inhibitors (Katoh et al., 2017). The anti-inflammatory activity of rivaroxaban was found to be associated with the inhibition of FXa-activated inflammatory responses. The expressions of ICAM-1 and IL-8 were increased when human atrial tissue sections were exposed to FXa. The combination of rapid pacing and FXa, which mimics AF, promoted significant upregulation of protease-activated receptor (PAR-1), PAR-2, ICAM-1, and IL-8. Rivaroxaban blocked the upregulation of PAR, ICAM-1, and IL-8. Taken together, these results suggest that FXa may mediate inflammatory signaling in atrial tissue by activating protease-activated receptors (Bukowska et al., 2013). Considering the evidence that FXa can bind to PARs and activate them (Steinberg 2005), the potential anti-inflammatory effect of rivaroxaban may be due to direct FXa inhibition.

It is widely accepted that inflammation and oxidative stress play important roles in the development of atrial fibrillation (Dudley et al., 2005; Neuman et al., 2007). Since the first report by Bruins et al., there has been increasing evidence that inflammatory conditions are closely related to the development of atrial fibrillation (Zhang et al., 2017; Zhou and Dudley 2020). Whether atrial fibrillation is a cause or a consequence of the inflammatory process, it is closely related to oxidative stress fixed by infiltration of the myocardium by inflammatory cells such as macrophages, accompanied by the release of reactive oxygen species (ROS) (Li et al., 2010). The inflammatory state leads to the activation of RAAS, which in turn activates NADPH oxidase. Thus, these processes trigger TGF-β pathway signaling and structural and electrical remodeling of the left atrium (Jalife 2014). An increased expression of various inflammatory cytokines and chemokines such as interleukin-1 and interleukin-6, tumor necrosis factor-alpha (TNF-α), or monocyte chemoattractant protein-1 (MCP-1) can be observed to have progression to chronic atrial fibrillation and recurrence of atrial fibrillation after cardioversion (Hu et al., 2015).

Our research found that HBG1 protein matched well with HBD and MASP1(Xa) protein surfaces, which was conducive to forming a stable binding effect. There exists a significant interaction between HBG1, HBD, and PT via the BP neural network and support vector machine. Enrichment analysis showed that HBG1 and HBD were mainly enriched in the blood coagulation and in the regulation of immune response. James et al. found that HBD is closely related to inflammation, and observed upregulation of HBD expression during infection and inflammation (Özdemir et al., 2020). Chen et al. found that HBD included oxygen transport, iron binding, coagulation, and binding to oxygen in GO annotations (Chen et al., 2020). HBG1 induces fetal hemoglobin (HbF) expression and reduces morbidity and mortality in hemoglobin disorders (Platt et al., 1994). The two β-hemoglobins produced by HBG1 and HBDβ-like globin genes may be involved in the occurrence and development of atrial fibrillation through inflammation and stress. Katayama et al. found that in patients with normal LV systolic function, increased LV mass index and low Hb concentrations were independently associated with LV enlargement (Katayama et al., 2010). This result suggests that hemodynamic changes in low or high hemoglobin levels can affect the development of LA remodeling and AF before overt changes such as LV hypertrophy or systolic dysfunction occur. It is further suggested that the two β-like globin genes, HBG1 and HBD, may directly affect the development of left atrial remodeling and atrial fibrillation and the state of blood coagulation by changing the level of hemoglobin or the inflammatory state. The blood lipids’ parameters (LDL-C, TC, and TG) were downregulated in the AF + edoxaban group. There were significant differences in blood lipid parameters (HDL-C, LDL-C, TC, and TG) between the AF group and the CON group. We consider the reason may be that changes in blood lipids cause increased blood viscosity, leading to poor coronary blood flow, which in turn induces the occurrence of AF; AF itself is an inflammatory reaction process, and inflammation affects the process of blood lipid metabolism. Since hyperlipidemia is a risk factor for other heart diseases, it appears that hyperlipidemia may also be a risk factor for AF. However, there is a “cholesterol paradox” in AF, and the association between lipid levels and the risk of new-onset AF is less clear. A number of recent epidemiological studies have explored the relationship between lipid levels and the risk of new-onset AF, with some studies showing no significant association, and some studies have shown that low blood lipid levels are associated with a lower risk of new-onset AF (Li et al., 2018; Yao et al., 2020).

The PT and APTT in the AF + edoxaban group and AF + rivaroxaban group were more increasing than in the AF mice (*p* < 0.05). AF is a hypercoagulable state. Through bioinformatics analysis in the previous stage, compared with the CON group, HBG1 and HBD were significantly increased in the AF group, while HBG1 and HBD were the main molecules involved in oxygen transport, and HBG1 and HBD had a significant protein interaction with factor Xa, resulting in a significant difference in coagulation function between the AF group and the CON group; Mariya Negreva et al. found that paroxysmal AF tends to have an early hypercoagulable state, involving intrinsic and extrinsic coagulation pathways in their study (Negreva et al., 2020). Rivaroxaban is a commonly used clinical anticoagulant. It specifically inhibits the activity of plasma factor Xa, thereby inhibiting the activity of prothrombin, preventing the activation of internal and extrinsic coagulation, reducing blood viscosity, and inhibiting the formation and expansion of thrombosis. It has high bioavailability, fast onset, and significant curative effect. It is widely used in acute coronary syndrome, stable coronary artery disease, thrombosis after atrial fibrillation, etc., which can greatly reduce the risk of adverse cardiac events (Carter et al., 2018).

The structure of cardiac muscle tissue in the AF + edoxaban group was better than that in the AF group and the AF + rivaroxaban group (*p* < 0.05). Compared with the AF group, the collagen fiber percentage in the AF + edoxaban group was decreased significantly (*p* < 0.05). Rivaroxaban has been shown to have endothelial protective and repairing properties, as the drug significantly enhanced HUVEC viability, growth, and wound healing. These effects may be mediated by the upregulation of u-PA and its enhanced functional activity. Rivaroxaban also counteracted the proinflammatory effects of FXa at the endothelial cell level, possibly through its direct inhibitory effect, and showed functional significance in inhibiting FXa-induced platelet adhesion to endothelial cells. More importantly, rivaroxaban appears to increase endothelial fibrinolytic pathway activity via u-PA activation, which together with its known anticoagulant activity will help create a global antithrombotic environment within the blood vessel (Álvarez et al., 2018).

There exist significant differences in blood lipids and coagulation between the AF + edoxaban group and the AF + rivaroxaban group. In the previous bioinformatics analysis, compared with the CON group, HBG1 and HBD were significantly increased in the AF group. HBG1 and HBD are the main molecules involved in oxygen transport. At the same time, HBG1 and HBD have significant protein interactions with factor Xa. Therefore, HBG1 and HBD affect the state of blood lipids and coagulation function. Our results show that edoxaban reduces HBG1/HBD more significantly, resulting in differences in blood lipids and coagulation indexes between the two groups.

However, this study also has certain shortcomings. First, we did not include edoxaban in clinical studies of patients with atrial fibrillation. Therefore, in future research, we will apply for the inclusion of edoxaban in clinical application, then detect the expression of HBG1 and HBD in the blood of patients with atrial fibrillation before and after taking edoxaban, and obtain more sufficient clinical evidence.

## Conclusions

Edoxaban might inhibit the expressions of HBG1 and HBD and then inhibit the inflammation, blood lipids, and fibrosis further. Furthermore, coagulation was inhibited, and fibrinolysis was activated at the effect of edoxaban. Finally, the development of AF to stroke was prevented. In addition, the effect of edoxaban was superior to rivaroxaban.

## Data Availability

The original contributions presented in the study are included in the article/Supplementary Material, further inquiries can be directed to the corresponding authors.
